# Potential chiral fluorescent molecular probes based on an α,β-unsaturated ketone for anion detection

**DOI:** 10.1038/s41598-019-55421-2

**Published:** 2019-12-11

**Authors:** Congshu Li, Lixia Liu, Weitong Pan, Yanmei Chen, Xuefang Shang, Yingling Wang, Tianyun Wang, Xiufang Xu

**Affiliations:** 1 Key Laboratory of Medical Molecular Probes, School of Basic Medical Sciences, Xinxiang Medical University, Xinxiang, Henan 453003 China; 2 Queen Marry University of London, Nanchang University, Nanchang, Jiangxi 330031 China; 3 Department of Biochemistry, Xinxiang Medical University, Jinsui Road 601, Xinxiang, Henan 453003 China; 4 Department of Chemistry, Nankai University, Tianjin, 300071 China

**Keywords:** Analytical chemistry, Inorganic chemistry, Supramolecular chemistry

## Abstract

A series of potential chiral compounds containing an α,β-unsaturated ketone was developed for anion detection. The interplay of compounds and biological momentous anions (Cl^−^, H_2_PO_4_^−^, I^−^, AcO^−^, HS^−^, F^−^, and Br^−^) was evaluated by UV-vis experiments, fluorescence experiments, and electrochemical tests. By comparison, compound 1 had the best selectivity and compound 5 had the strongest binding ability among the five compounds. And compound 5 had the highest sensitivity to H_2_PO_4_^−^ among the measured anions, and it also can be applied to actual samples, the content of H_2_PO_4_^−^ tested in the potassium dihydrogen phosphate fertilizer solution reached above 97.5% of the marked content, and the recovery rates were within the range of 98.5–99.1%, attesting that this method was reliable for the test of H_2_PO_4_^−^ in fertilizer. Through HRMS titration, circular dichroism and optical rotation experiments, the probable interacted mechanism was proved that the interaction site was the C=C of the α,β-unsaturated ketone structure. In addition, the interacted mechanism was researched from the perspective of chirality. Furthermore, theoretical investigation analysis was introduced to reveal that the roles of molecular frontier orbitals in molecular interplay were determined. Therefore, this series of potential chiral compounds has potential application prospects in anion recognition.

## Introduction

Chirality is a universal feature in the universe, which is reflected in the emergence and evolution of life^1–3^. With the continuous in-depth research on molecular recognition, chiral molecular recognition has become an important topic^4–6^. At the same time, with the increasing attention on the safety and environmental problems with using radioactive probes and the advantages of high sensitivity and good selectivity, fluorescent probes have been widely used in molecular recognition. In view of the important role of anions (such as AcO^−^, HS^−^, F^−^, H_2_PO_4_^−^ etc.) in nature, a great deal of effort has been made within the field of supramolecular chemistry to devise and synthesize receptors capable of selectively identifying anions^7–10^. As a consequence, the development of novel molecular probes for anion sensors has been proven to be fairly active research domain^11–21^. However, there are few reports on potential chiral fluorescent molecular probes used to detect anions. Therefore, it is very important to use potential chiral fluorescent molecular probes to selectively detect anions.

Normally, an α,β-unsaturated ketone is linked to fluorescent groups, which will result in weak or almost no fluorescence response of fluorescent probes due to suppression of photo-induced electronic transfer (PET). The nucleophilic additive reaction of anions with an α,β-unsaturated ketone blocks the PET process and causes fluorescence recovery. Thus, we can detect the anions that we want^22,23^.

**Scheme 1:**
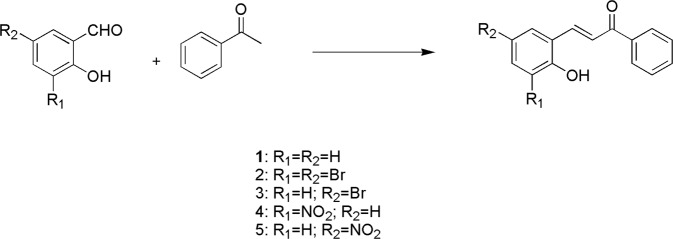
The route of synthesized compounds.

In this thesis, a string of potential chiral compounds containing an α,β-unsaturated ketone structure was designed and synthesized (Scheme 1). The synthetic compounds displayed a high combination capacity for AcO^−^, HS^−^, F^−^, H_2_PO_4_^−^ in the sensing of different anions by UV-vis, fluorescence, electrochemical, circular dichroism and optical rotation experiments. Fortunately, the crystal of compound 1 was also obtained, which provided the foundation for the host-guest interaction.

## Results and Discussion

### X-ray crystallography

The ethanol solution of compound 1 was placed so that the ethanol evaporated slowly and the single light-yellow crystal was separated. The determination of its crystal structure was carried out using X-ray diffraction analysis (see Supplementary Fig. S1). X-ray diffraction data for 1 was collected, and the structural refinement results were shown in Table S1. As shown in Fig. S2, hydrogen bonds were formed between two molecular compounds, which formed a “T” configuration. The hydrogen bonds formed were linked by O(1)-H(1)…O(2)#1. (see Supplementary Table S2 Symmetry transformations used to generate equivalent atoms: #1 −x +5/2, y + 1/2, −z + 1/2). Additionally, π-π stacking of different “T” configurations formed a stratified structure.

### UV-vis titration

**Figure 1:**
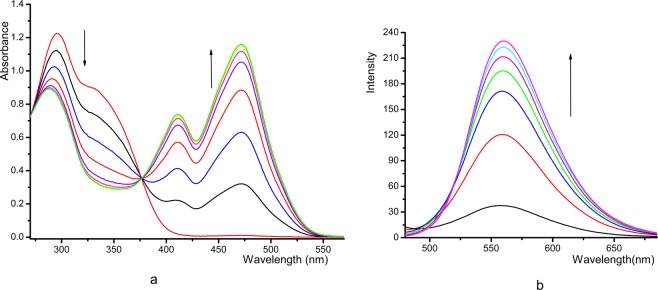
UV-vis spectral (**a**) and fluorescence (**b**) changes of compound **5** (4 × 10^–5^ mol^.^L^−1^) upon the addition of H_2_PO_4_^−^. All spectra were recorded in DMSO solution, (**a**) H_2_PO_4_^−^: ((0–8) × 10^−5^ mol·L^−1^); (**b**) H_2_PO_4_^−^: (0–20.8) × 10^−5^ mol·L^−1^), λ_ex_ = 412 nm. Arrows indicated the direction of increasing anion concentration.

The combining capacities of probes (1–5) with anions (H_2_PO_4_^−^, AcO^−^, HS^−^, F^−^, Cl^−^, I^−^ and Br^−^) in DMSO solution were investigated via UV-vis spectroscopy. Results manifested that these compounds (1–5) showed different binding abilities for anions. As seen in Figs. S3a, S4, compound 1 took on two intense absorption bands at at approximately 292 and 365 nm, while the remarkable absorption band gradually arose at approximately 500 nm with the gradual addition of F^−^ and H_2_PO_4_^−^. Simultaneously, the strengths of the absorption peaks at 292 and 365 nm decreased. A distinct isoabsorptive point was noted at 376 nm and it turned out that 1 and H_2_PO_4_^−^ formed a stable complex in a certain proportion. The binding of H_2_PO_4_^−^, AcO^−^, HS^−^, F^−^ to compound 4, and H_2_PO_4_^−^, Aco^−^, F^−^ and HS^−^ to 5 produced phenomenona similar to that of H_2_PO_4_^−^ to 1 (see Fig. 1a, Supplementary Figs. S3d, S7, S8). However, the excessive addition of else anions induced only the faint changes and even could be overlooked in the UV-vis spectra. For compounds 2 with two electron-withdrawing groups (*-Br*), it had two peaks at 320, 360 nm. After the addition of H_2_PO_4_^−^, AcO^−^ and F^−^, the peak increased at 320 nm, decreased at 360 nm, and formed a new peak at 500 nm, forming isoabsorptive points at 330 and 410 nm. (see Supplementary Figs. S3b, S5). Compounds 3 with H_2_PO_4_^−^, AcO^−^, HS^−^, F^−^ showed the same occurrences, while the other ions showed little change (see Supplementary Figs. S3c, S6). Additionally, the absorption intensity of compound 2 (3,5-dibromosalicylaldehyde derivative) was stronger than that of compound 3 (5-bromosalicylaldehyde derivative). This was due to the fact that two -Br have more electron-withdrawing property than one -Br. The binding spectra of probes 1, 4, 5 and anion were different from that of 2, 3 due to the different substituents. In comparison, compound 5 had the largest spectral change in combination with anions among the five compounds, followed by 2, possibly because -NO_2_ showed stronger electron-withdrawing property than -Br, which was conducive to nucleophilic addition, and increased the combining ability of C=C in compounds and ions. While the -NO_2_ located in the 3-position of the salicylaldehyde was weaker than that of the 5-position of the salicylaldehyde, which may be due to steric hindrance.

### Fluorescence response

The interactions of detected anions and compounds (1–5) can also be studied by changes in fluorescence before and after the reaction. The free compound 1 exhibited a peak at 492 nm. When different anions including H_2_PO_4_^−^ (see Supplementary Fig. S9a), F^−^ (see Supplementary Fig. S10a), and AcO^−^ (see Supplementary Fig. S10b) were titrated into a DMSO solution of compound 1, its fluorescence intensity progressively strengthened at 600 nm, and a fresh absorption peak appeared. When excessive Cl^−^, Br^−^, I^−^ and HS^−^ ions were added, there was almost no influence on the fluorescence intensity. The fluorescence spectral response of compound 5 was so weak that it can be almost ignored. Then, the fluorescence band with 560 nm as the center was heightened with the dripping of H_2_PO_4_^−^ (see Fig. 1b), F^−^ (see Supplementary Fig. S12a), AcO^−^ (see Supplementary Fig. S12b), and HS^−^ (see Supplementary Fig. S12c) into compound 5. Similar changes in the fluorescence spectra occurred in the addition of H_2_PO_4_^−^ (see Fig. S9a), F^−^ (see Supplementary Fig. S11a), AcO^−^ (see Supplementary Fig. S11b), and HS^−^ (see Supplementary Fig. S11c) to compound 2 and H_2_PO_4_^−^ to compounds 3 (see Supplementary Fig. S9c) and 4 (see Supplementary Fig. S9d). The additions of other anions caused little or no change and therefore can be ignored. Two possible mechanisms to account for the above fluorescence enhancement were exploited: (1) suppression of PET and (2) the rigidity of the host molecule induced by the binding guest^24,25^. Free compounds exhibited a weak fluorescence response, and the reason might be that without the additions of anions, the hydrogen atom on the hydroxyl group (–OH) of the free compound could form intermolecular hydrogen bonds with the oxygen atom of the α,β-unsaturated ketone (see Supplementary Fig. S2), leading to photoinduced electron transfer. However, electrons were shifted from the compound to the fluorophore when the anion was added to the compound solution, which led to enhancement of the emission intensity.

### Binding constants

The complexes were obtained by combining the compounds (1–5) with anions in a proportion of 1:1 through the analysis of the Job plots. The binding constants derived from the non-linear square method using UV-vis titration were shown in Table 1^26,27^. From Table 1, compounds (2–5) with electron-withdrawing group showed the following binding ability sequence: H_2_PO_4_^−^ > F^−^ > AcO^−^ > HS^−^ > Cl^−^, I^−^, Br^−^. The reason may be that (1) nucleophilicity of anion; (2) spatial matching between probes and anions; (3) hydrogen bonding between host and guest. The tetrahedral space configuration of H_2_PO_4_^−^ had a higher spatial fit with the compounds, and it can form hydrogen bonds with the probes^24,25^. The reason why compound 5 had the strongest ability to bind H_2_PO_4_^−^ might be that as an electron-withdrawing group, the 5-position nitro group enhanced the combining capacity of the C=C of compound 5 with H_2_PO_4_^−^. However, compound 1 without electron-withdrawing group showed high sensitivity for F^−^ with spherical structure in different anions, which may be related to steric hindrance. It also can be found from the data in the Table 1 that electron-withdrawing groups could enhance the binding ability of compounds with anions.

**Table 1:** Binding constants of synthesized compounds with various anions.

Anion^a^	HS^−^	F^−^	AcO^−^	H_2_PO_4_^−^	Cl^−^, Br^−^ I^−^
*K*s_1_	ND^b^	(2.29 ± 0.66) × 10^4c^	ND^b^	(1.87 ± 0.08) × 10^2c^	ND^b^
*K*s_2_	(3.84 ± 1.25) × 10^2c^	(3.61 ± 0.51) × 10^3c^	(3.25 ± 0.11) × 10^3c^	(1.53 ± 0.18) × 10^4c^	ND^b^
*K*s_3_	ND^b^	(9.80 ± 2.75) × 10^3c^	(5.44 ± 0.71) × 10^3c^	(1.43 ± 0.12) × 10^4c^	ND^b^
*K*s_4_	(1.15 ± 0.07) × 10^3c^	(3.50 ± 0.22) × 10^3c^	(1.59 ± 0.11) × 10^3c^	(8.09 ± 0.38) × 10^3c^	ND^b^
*K*s_5_	(2.51 ± 0.50) × 10^3c^	(1.07 ± 0.61) × 10^3c^	(2.96 ± 0.74) × 10^3c^	(4.00 ± 0.13) × 10^5c^	ND^b^

^a^Anions was added in the form of sodium sulfide or tetra-*n*-butylammonium salts.

^b^The spectra changed little and the binding constant could not be determined.

^c^The binding ratio is 1:1.

**Figure 2:**
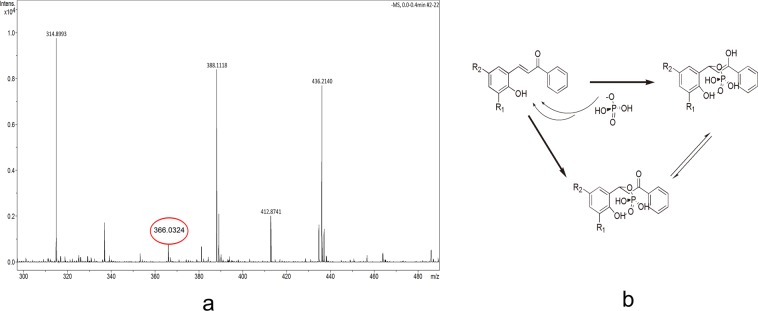
(**a**) ESI-HRMS spectrum of compound **5** after addition of H_2_PO_4_^−^ in DMSO solution. HRMS(ESI) (m/z):366.0324 (M-H)^−^; and (**b**) interaction mechanism between H_2_PO_4_^−^ and the compounds.

Taking H_2_PO_4_^−^ as example, HRMS (ESI) was used to study the mechanism of compounds binding to anions. As shown in Fig. 2a, a new molecular ion peak appeared at m/z = 366.0324 (M-H)^−^ (5-H_2_PO_4_^−^) after adding H_2_PO_4_^−^ to compound 5. Considering the above work, the reaction mechanism of the compounds and H_2_PO_4_^−^ may have two forms: (1) the nucleophilic reaction of H_2_PO_4_^−^ and the electron-deficient C=C; and (2) hydrogen bond formation between the –OH of salicylaldehyde and H_2_PO_4_^−^ (Fig. 2b)^10,11^.

### Detection limit

As compound 5 had the strongest binding ability, so taking it for example, the linear relationship between compound 5 and H_2_PO_4_^−^, F^−^, HS^−^, AcO^−^ in DMSO solution was determined by UV-vis titration. The absorption value of compound 5 (4 × 10^–6^ mol·L^−1^) at 470 nm was 0.049, the molar absorption coefficient was calculated as 31005 L· mol^−1^ ·cm^−1^. As can be seen from the Figure S13a, when the concentration of AcO^−^ varies within the range of 0–10^−2^ mol·L^−1^, the UV-vis absorption value of compound 5 had a linear relationship with the concentration of AcO^−^, and the correlation coefficient was above 0.99. The absorption value of compound 5 risied with the addition of AcO^−^, until the concentration of AcO^−^ increased to 7.36 × 10^–3^ mol·L^−1^, the absorption value of compound 5 rised to 1.3 times^28^, which means the detection limit of compound 5 to AcO^−^ was 7.36 × 10^–3^ mol·L^−1^. With the same method and the same conditions, the detection limit of 5 to HS^−^ was 8 × 10^–5^ mol·L^−1^(see Supplementary Fig. S13b), that of F^−^ was 4.8 × 10^–5^ mol·L^−1^(see Supplementary Fig. S13c), and that of H_2_PO_4_^−^ was 1.2 × 10^–5^ mol·L^−1^(see Supplementary Fig. S13d). According to the above data, the order of the anions sensitivity of the compound 5 was H_2_PO_4_^−^ > F^−^ > HS^−^ > AcO^−^.

### Electrochemical experiment

**Figure 3:**
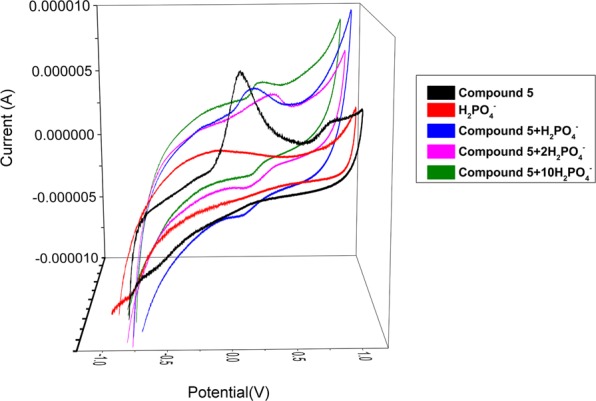
Cyclic voltammetry of compound **5** and Compound **5**-H_2_PO_4_^−^.

In order to further explore the synthesized compound as anion probe, compound 5 was taken as an example to conduct cyclic voltammetry study in DMSO. Saturated sodium chloride solution was used as the electrolyte solution, a platinum electrode was the assistant electrode, and a calomel electrode was the reference electrode. In addition, a glassy carbon electrode was selected as the working electrode. The scan range was −1 V to 1 V, and the scanning speed was set to 50 mV·s^−1^. Figure 3 showed the cyclic voltammetry behavior when compound 5 interacted with H_2_PO_4_^−^. At 0.8 V, compound 5 had a weak oxidation peak, while it had an obvious oxidation peak at 0.1 V. After adding H_2_PO_4_^−^, the oxidation peak of 0.8 V disappeared, and there was a new reduction peak near 0.1 V. Moreover, as the concentration of H_2_PO_4_^−^ increased, the anodic peak and the cathodic peak potentials both moved in the positive direction, and the current gradually decreased. After HS^−^ was added, the oxidation peaks disappeared and a weak reduction peak appeared at −0.6 V. And the current decreased gradually (see Supplementary Fig. S14a). With the addition of AcO^−^ (see Supplementary Fig. S14b) and F^−^ (see Supplementary Fig. S14c), the oxidation peak of 0.8 V disappeared, the oxidation peak at 0.1 V moved in a negative direction, and a new reduction peak appeared near 0.4 V, and the peaks currents decreased with the increasing of anions concentration. The changes in the figures were explained by the formation of complexes between compound 5 and AcO^−^, F^−^, H_2_PO_4_^−^. The oxidation peak of compound 5 at 0.8 V may be the characteristic peak of α,β-unsaturated ketone, and the peak at 0.1 V may be the characteristic peak of -NO_2_. As compound 5 reacted with AcO^−^, F^−^, and H_2_PO_4_^−^, new redox systems were formed, the redox reaction of compound 5 gradually weakened, and the α,β-unsaturated ketone system was destroyed, so the oxidation peak at 0.8 V disappeared. The addition of Cl^−^, Br^−^, and I^−^ caused almost no change in the redox signal intensity of compound 5 (see Supplementary Fig. S14d). This indicated that there was little electrochemical reaction between Cl^−^, Br^−^ or I^−^ and compound 5^29^.

### Circular dichroism

**Figure 4:**
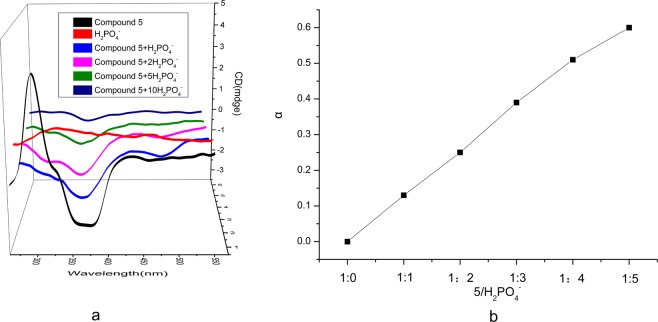
(**a**) The circular dichromatic spectra of H_2_PO_4_^−^, compound **5**, compound **5** toward the addition of 1, 2, 5, 10 equiv. H_2_PO_4_^−^ in DMSO (**b**)the change in optical rotation of compound **5** after binding with H_2_PO_4_^−^ in DMSO.

Circular dichroism is a new method for studying potential chiral and stereoscopic structures of molecules, but it is seldom used in anion detection. Therefore, compound 5 was taken as an example to verify the interaction mechanism between compound and anions from the perspective of chirality through circular dichroism experiment. As shown in Fig. 4a, in DMSO, H_2_PO_4_^−^ was achiral, and thus, it had no circular dichroism absorption. However, compound 5 had a positive characteristic absorption peak at 285 nm and a negative peak at 360 nm. This was due to^30,31^ the fact that when a vinyl group was conjugated with a carbonyl group, the n → π^*^ transition of the carbonyl group occurred in the long-wave direction at 320–360 nm, at the same time, a strong π → π^*^ band appeared at 240 nm. According to Woodward’s rules and related to the environment of the chromophore, the circular dichromatic peak of compound 5 in the Fig. 4a appeared. When compound 5 reacted with H_2_PO_4_^−^, the structure of α,β-unsaturated ketone was destroyed. Therefore, with the continuous addition of H_2_PO_4_^−^, the peak at 285 nm disappeared and the peak strength at 360 nm decreased. The same phenomenon occurred with the addition of HS^−^, AcO^−^ and F^−^ (see Supplementary Fig. S15). But, in methanol solvent, compound 5 had a positive peak at 290 nm, and when it combined with ions, the positive peak disappeared. This may be due to α,β-unsaturated ketone keto-enol tautomerism^24,25^ and solvent effect. In DMSO, there were intermolecular hydrogen bonds between two molecules (see Supplementary Fig. S2), so the main form of existence was ketone. However, as methanol is a protic solvent, it is easy to form hydrogen bonds with hydroxyl groups in the compound, which destroyed the original hydrogen bonds. Therefore, the main form of existence may be enol form. So the carbonyl peak at 360 nm vanished. As a result, the interaction of compound 5 and ions had different circular dichroism spectra in methanol and DMSO. The above outcome confirmed that the action site was the C=C of the α,β-unsaturated ketone structure, which verified the mechanism shown in Fig. 2b above.

### Specific rotation

Again, take the reaction of compound 5 with H_2_PO_4_^−^, we did the optical rotation experiment to verify further. In the DMSO, the value of [α]_5_ was 0.0 (0.004 mol·L^−1^ in DMSO), when compound 5 was combined with H_2_PO_4_^−^, the optical rotation increased continuously. When H_2_PO_4_^−^ was added to five equiv., [α]_5-H2PO4_^−^ = 0.6 (0.004 mol·L^−1^ in DMSO)(Fig. 4b). Combined with the circular dichroism spectra, this may be due to that the positive and negative peak intensities of compound 5 were almost identical and cancel each other out, so the optical rotation was 0. When H_2_PO_4_^−^ was added, only the absorption peak of carbonyl group was left, so it had optical rotation. When methanol was the solvent, the value of [α]_5_ was 0.6 (0.004 mol·L^−1^ in CH_3_OH). When the added H_2_PO_4_^−^ was 1:1 with compound 5, the value of [α]_5-H2PO4_^−^ was 0.5 (0.004 mol·L^−1^ in CH_3_OH), When the ratio was 1: 5, the value of [α]_5-H2PO4_^−^ was 0.0 (0.004 mol·L^−1^ in CH_3_OH), The optical activity disappeared (see Supplementary Fig. S16). The reaction of compound 5 and H_2_PO_4_^−^ in methanol and DMSO had opposite results due to solvent effect. The specific reasons have been explained in the circular dichroism section. The experiments confirmed the above conclusion of circular dichroism experiment.

### Theoretical investigation

**Figure 5:**
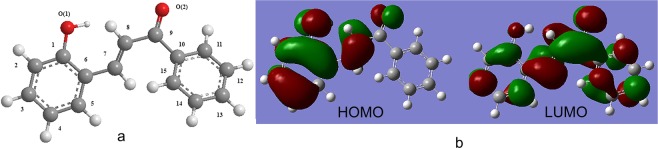
(**a**) The geometry of compound **1** and (**b**) Molecular orbital level of compound **1**.

In order to obtain further structural information, the calculation of compound 1 was implemented in Gaussian 03. Its geometric configuration optimization (Fig. 5a) was accomplished by density functional theory combined with the B3LYP exchange correlation function and 3–21 G basis set^32^. Comparison of bond lengths (see Supplementary Table S3) and angles (see Supplementary Table S4) between the X-ray diffraction crystal structure and theoretically optimized model structure of compound 1 shows that the two data sets were basically consistent. The differences in the data may be caused by the fact that most optimizations were obtained under the gas phase conditions, and the difference in the base group and method used for calculation could be neglected. Therefore, this manifested the veracity of the theoretical method^33,34^. Meanwhile, we could know that 1 had no intramolecular hydrogen bonds. The literature^35,36^ stated clearly that intramolecular hydrogen bonding and electron-withdrawing group can heighten the anion binding ability. Therefore, this could explain why compound 1 had a very weak anionic binding capacity, which also could be proven by the UV-vis and fluorescence experiments.

In addition, Gaussian 03 not only optimized the computational structure of compound 1 but also obtained its molecular frontier orbitals (Fig. 5b). Its lowest unoccupied molecular (LUMO) level and highest occupied molecular (HOMO) level made it possible for electron transition. For compound 1, the density in the HOMO orbital was primarily concentrated in the salicylaldehyde part; nevertheless, the electron density in the LUMO was distributed throughout the whole molecule. Thus, the change caused by the combination of host and guest in the UV-vis spectra can be explained by the electron transition of the HOMO^37^.

### Application in sample

Fluorescent probes were applied to real samples^38^. Under the optimal conditions, taking the concentration range of 0–1.0 mg·mL^−1^ of H_2_PO_4_^−^ as an example, the UV-vis absorption of compound 5 was linearly correlated with H_2_PO_4_^−’^s concentration(see Supplementary Fig. S17), and the correlation coefficient was 0.9945. The linear regression equation was A = 0.15414C (mg·mL^−1^) +0.2801 (n = 8). The 2.0 g potassium dihydrogen phosphate fertilizer (main content was 1.96 g) was dissolved in 50 mL DMSO and the precipitation was filtered out. The filtrate was then diluted to a main content of 0.392 mg·mL^−1^. The measured results using the above methods were shown in Table S5. Through the three measurements, the content of H_2_PO_4_^−^ tested in the fertilizer solution reached above 97.5% of the marked content. It can be seen that there was no significant difference between the content of marker and the result obtained by this method. Recovery experiments were performed on each analyzed samples by adding 0.5 mg·mL^−1^ of H_2_PO_4_^−^ standard. As shown in the Table S5, the recovery rates were within the range of 98.5–99.1%, attesting that this method was reliable for the test of H_2_PO_4_^−^ in fertilizer. The results demonstrated the applicability of the probe to actual samples.

## Methods

The vast majority of raw materials were gained commercially, and all reagents and solvents were analytical reagents. HS^−^ was from NaHS, and other anions used were from the tetrabutylammonium salts purchased from Aladdin (Shanghai, People’s Republic of China) and did not need to be purified. The solvent dimethyl sulfoxide (DMSO) was gained by reduced pressure distillation after drying by CaH_2_. ^1^H-NMR information was obtained by a Unity Plus-400 M*Hz* spectrometer (Bruker, Massachusetts, USA). C, H, and N elements were analyzed by a Vanio-EL elemental analyzer (Elementar, Philadelphia, PA, USA). Electrospray Ionization with High-Resolution Mass Spectrometry (HRMS (ESI)) was achieved using a Mariner apparatus(Bruker, Massachusetts, USA). At 298 K, an Eclipse fluorescence spectrophotometer (Agilent, State of California, USA) was used for fluorescence experiments; circular dichroism data was measured through a Chirascan (Applied Photophysics, Surrey, UK); an UV2600 UV-vis spectrophotometer (Shimadzu, Kyoto, Japan) was used to perform UV-vis titration. A VersaSTAT 3 Potentiostat Galvanostat (Princeton Applied Research (Ametek), New Jersey, USA) was used for electrochemical experiments. The binding constants (*K*_s_) were acquired by nonlinear least squares (curve fitting)^39–41^. Five compounds (1–5) were synthesized in light of following procedures.

Synthesis of compound 1: Salicylaldehyde (4 mmol, 0.4966 g) and acetophenone (2 mmol, 0.2515 g) were added to the flask and ethanol solution (15 mL) was poured in to dissolve them. Then, the pale-yellow solution was obtained. Four milliliters of 40% (m/v) NaOH solution was added into the mixed solution. The solution developed into yellow with the first drop of NaOH, and then the yellow color deepened stepwise with the continuous dripping of NaOH. After HCl (5%, 3.5 mL, m/v) was used to adjust the pH of the solution to 6, a yellow solid formed and was filtered at normal temperature. Next, it was dried in a vacuum environment after washing with high purity water and ethanol. Yield: 74%. m.p. 162–163 °C. ^1^H NMR (400 M*Hz*, DMSO) δ 10.31 (s, 1 H), 8.08 (dd, *J* 18.7, 11.5 *Hz*, 3 H), 7.87 (dd, *J* 11.1, 7.6 *Hz*, 2 H), 7.66 (d, *J* 7.3 *Hz*, 1 H), 7.57 (t, *J* 7.5 *Hz*, 2 H), 7.28 (t, *J* 7.7 *Hz*, 1 H), 6.94 (d, *J* 7.5 *Hz*, 1 H), 6.88 (t, *J* 7.4 *Hz*, 1 H) (see Supplementary Fig. S18). Elemental analysis: Calc. for C_15_H_12_O_2_: C, 80.34; H, 5.39; Found: C, 80.32; H, 5.39. HRMS (ESI) *(m/z)*: 247.0731 (*M* + Na)^+^ (see Supplementary Fig. S19).

Compounds (2–5) were made in an analogous manner.

Compound 2: Yield: 78%. m.p. 171–173 °C. ^1^H NMR (400 M*Hz*, DMSO) δ 10.28 (s, 1 H), 8.25 (d, *J* 2.3 *Hz*, 1 H), 8.19 (d, *J* 7.2 *Hz*, 2 H), 8.01 (d, *J* 2.6 *Hz*, 2 H), 7.83 (d, *J* 2.3 *Hz*, 1 H), 7.69 (t, *J* 7.3 *Hz*, 1 H), 7.58 (t, *J* 7.6* Hz*, 2 H) (see Supplementary Fig. S20). Elemental analysis: Calc. for C_15_H_10_Br_2_O_2_: C, 47.16; H, 2.64; Found: C, 80.32; H, 5.39. HRMS (ESI) *(m/z)*: 382.9111 (*M* + H) ^+^ (see Supplementary Fig. S21).

Compound 3: Yield: 75%. m.p. 163–165 °C. ^1^H NMR (400 M*Hz*, DMSO) δ 10.60 (s, 1 H), 8.19–8.14 (m, 3 H), 7.97 (s, 2 H), 7.63 (dt, *J* 15.1, 7.3 *Hz*, 3 H), 7.42 (dd, *J* 8.7, 2.5 *Hz*, 1 H), 6.90 (d, *J* 8.7 *Hz*, 1 H) (see Supplementary Fig. S22). Elemental analysis: Calc. for: C_15_H_11_BrO_2_: C, 60.40; H, 4.43; Found: C, 60.39; H, 4.43. HRMS (ESI) *(m/z)*: 300.9869 (*M*-H)^−^ (see Supplementary Fig. S23).

Compound 4. Yield: 68%. m.p. 117–119 °C. ^1^H NMR (400 M*Hz*, DMSO) δ 11.05 (s, 1 H), 8.39 (d, *J* 7.7 *Hz*, 1 H), 8.15 (d, *J* 7.4 *Hz*, 2 H), 8.12–8.05 (m, 2 H), 8.01 (d, *J* 15.8 *Hz*, 1 H), 7.70 (t, *J* 7.3 *Hz*, 1 H), 7.59 (t, *J* 7.6 *Hz*, 2 H), 7.15 (t, *J* 8.0 *Hz*, 1 H) (see Supplementary Fig. S24). Elemental analysis: Calc. for: C_15_H_11_NO_4_: C, 66.91; H, 4.12; N, 5.20; Found: C, 66.92; H, 4.12; N, 5.20. HRMS (ESI) *(m/z)*: 268.0615 (*M*-H)^−^ (see Supplementary Fig. S25).

Compound 5. Yield: 72%. m.p. 210–212 °C. ^1^H NMR (400 *MHz*, DMSO) δ 11.93 (s, 1 H), 8.84 (s, 1 H), 8.19 (d, *J* 8.2 *Hz*, 3 H), 8.12 (d, *J* 15.8 *Hz*, 1 H), 8.00 (d, *J* 15.8 *Hz*, 1 H), 7.69 (t, *J* 7.1 *Hz*, 1 H), 7.59 (t, *J* 7.6 *Hz*, 2 H), 7.12 (d, *J* 9.1 *Hz*, 1 H) (see Supplementary Fig. S26). Elemental analysis: Calc. for: C_15_H_11_NO_4_: C, 66.91; H, 4.12; N, 5.20; Found: C, 66.92; H, 4.12; N, 5.20. HRMS (ESI) *(m/z)*: 268.0617 (*M*-H)^−^ (see Supplementary Fig. S27).

## Conclusion

In summary, we developed five potential chiral molecular probes with “OFF-ON” fluorescence response for AcO^−^, HS^−^, F^−^, H_2_PO_4_^−^ detection. In addition, from HRMS and chiral means indicated that the binding site of the probe to the anion was on the α,β-unsaturated ketone structure. By comparison, compound 1 had the best selectivity and compound 5 had the strongest binding ability among the five compounds. And compound 5 had the highest sensitivity to H_2_PO_4_^−^ among the measured anions, and it also can be applied to actual samples.

## Supplementary information

Supplementary Information
