# Supplementary material for: Potential chiral fluorescent molecular probes based on an α,β-unsaturated ketone for anion detection
Source: Sci Rep. 2019 Dec 11;9:18838. doi: 10.1038/s41598-019-55421-2 (PMC6906485; doi:10.1038/s41598-019-55421-2)
Supplement: Supplementary file 1 — Supplementary Information [file 41598_2019_55421_MOESM1_ESM.pdf]

Potential chiral fluorescent molecular probes based on an α,β-unsaturated ketone for anion detection

Congshu Li,[a] Lixia Liu,[a] Weitong Pan,[b] Yanmei Chen,[a] Xuefang Shang,[a*] Yingling Wang[a], Tianyun Wang,[c] Xiufang Xu[d]

[a]Key Laboratory of Medical Molecular Probes, School of Basic Medical Sciences, Xinxiang Medical University, Xinxiang, Henan 453003 China

[b]Queen Marry University of London, Nanchang University, Nanchang, Jiangxi 330031 China

[c]Department of Biochemistry, Xinxiang Medical University, Jinsui Road 601, Xinxiang, Henan 453003 China

[d]Department of Chemistry, Nankai University, Tianjin, 300071, China

[*]Corresponding author: Tel +86-373-3029128, Fax +86-373-3029959

E-mail: <xuefangshang@126.com>

Figure

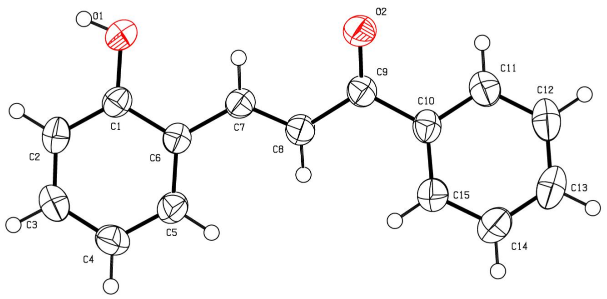

Figure S1. R-temp view of compound **1**

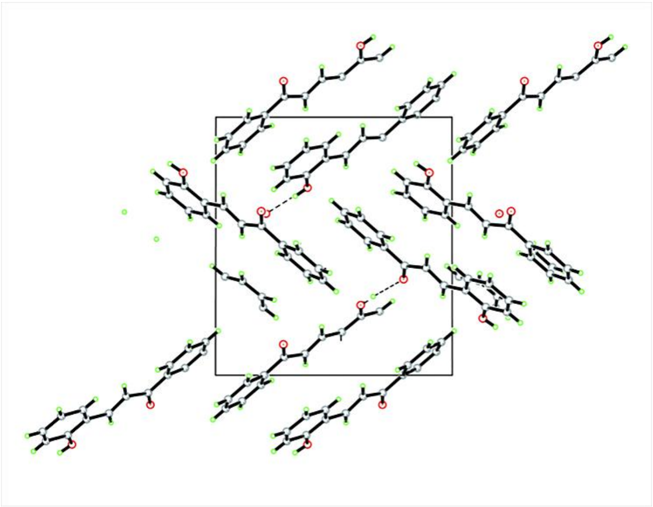

Figure S2. Crystal packing of compound **1** along the *a* axis.

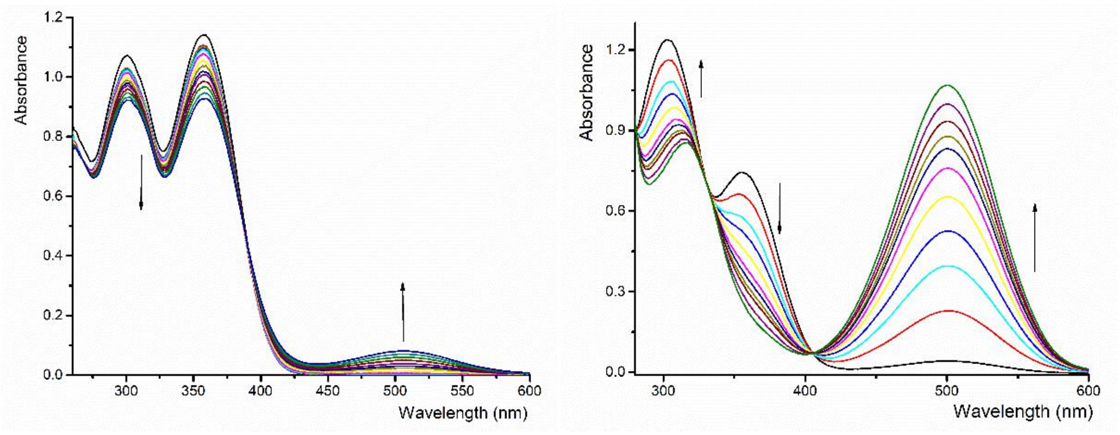

(a) (b)

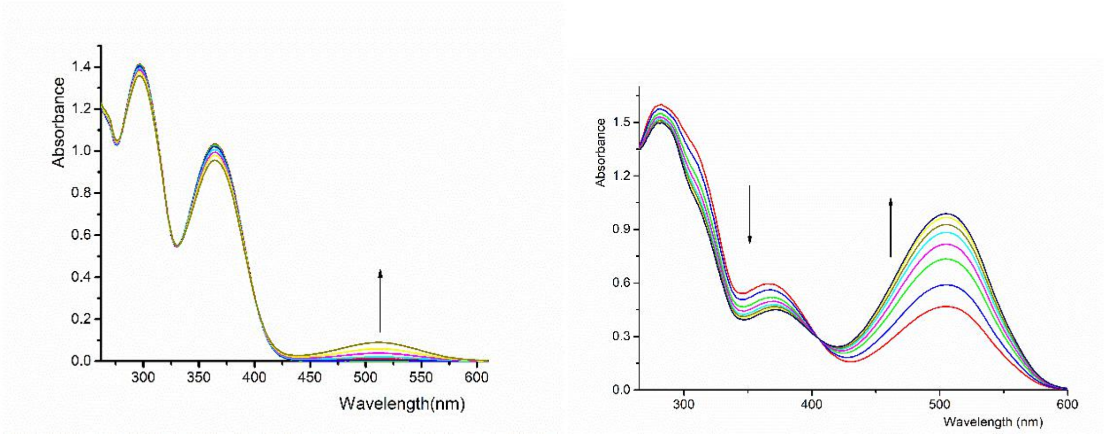

(c) (d)

Figure S3.UV-vis spectral changes of five compounds (4×10^{-5}mol·L^{-1}) upon the addition of $H_2PO_4^-$. All spectra were recorded in DMSO solution, a) **1**, $H_2PO_4^-$: ((0-192)$\times10^{-5}$ mol·L^{-1}); b) **2**, $H_2PO_4^-$: ((0-13.5) $\times10^{-5}$ mol·L^{-1}); c) **3**, $H_2PO_4^-$: ((0-8)$\times10^{-5}$ mol·L^{-1}); d) **4**, $H_2PO_4^-$: ((0-6.4)$\times10^{-5}$ mol·L^{-1}); arrows indicated the direction of increasing anion concentration.

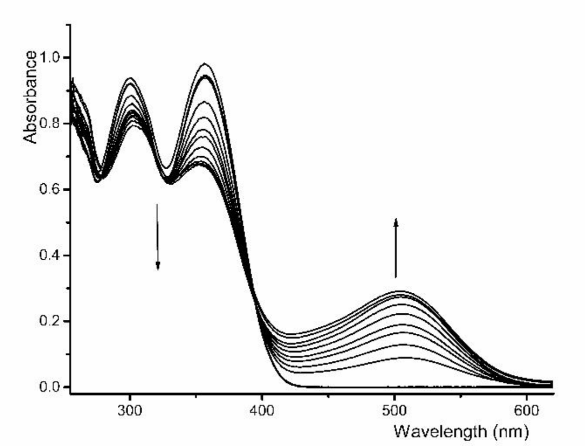

Figure S4. UV-vis spectral changes of compound **1** (4×10^{-5}mol·L^{-1}) upon the addition of F^- ((0-75.2)$\times10^{-5}$ mol·L^{-1}. Arrows indicated the direction of increasing anion concentration.

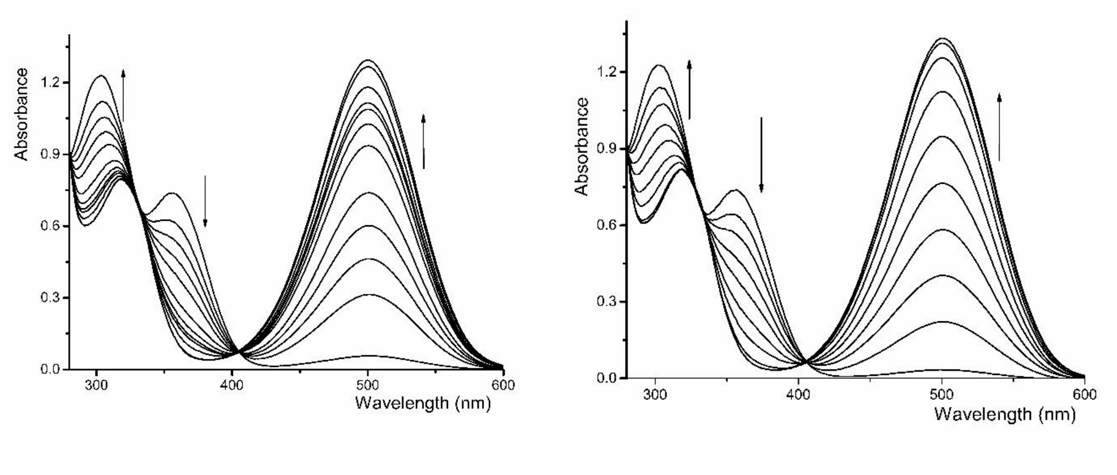

(a) (b)

Figure S5. UV-vis spectral changes of compound **2** (4×10^{-5}mol·L^{-1}) upon the addition of various anions. a) F^{-} ((0-12.8)×10^{-5} mol·L^{-1}, b) AcO^{-} ((0-8.0)×10^{-5} mol·L^{-1}. Arrows indicated the direction of increasing anion concentration.

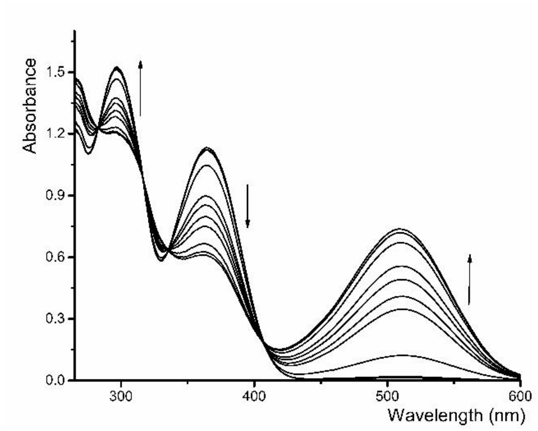

(a)

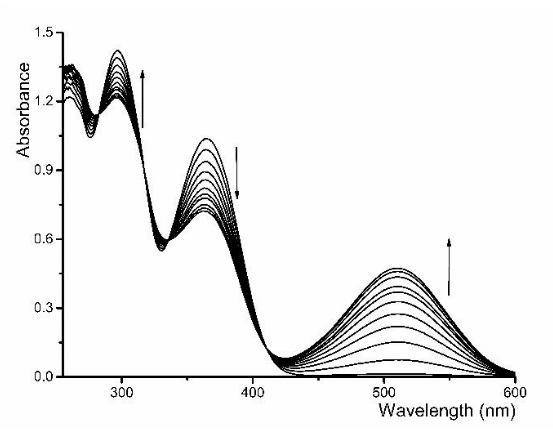

(b)

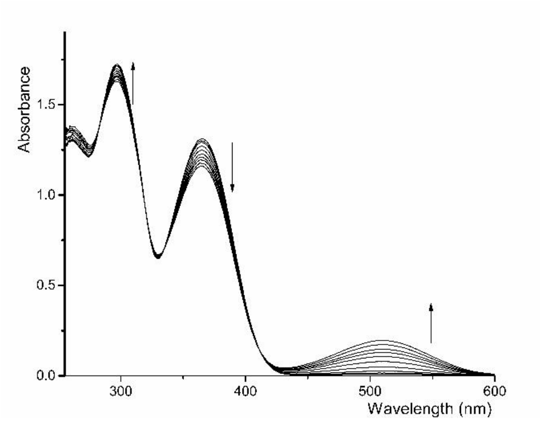

(c)

Figure S6. UV-vis spectral changes of compound **3** (4×10^{-5}mol·L^{-1}) upon the addition of various anions. a) F^{-} ((0-9.6)×10^{-5} mol·L^{-1}, b) AcO^{-} ((0-6.4)×10^{-5} mol·L^{-1}, c) HS^{-} ((0-20.8)×10^{-5} mol·L^{-1}. Arrows indicated the direction of increasing anion concentration.

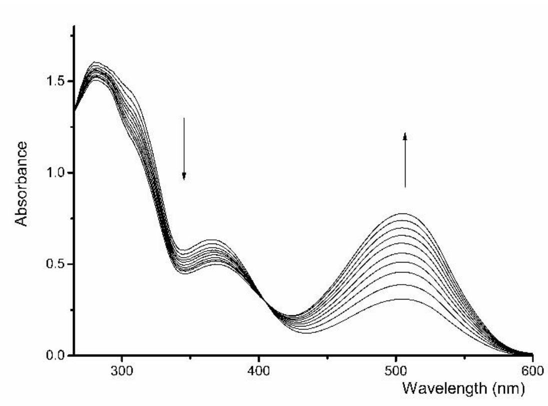

(a)

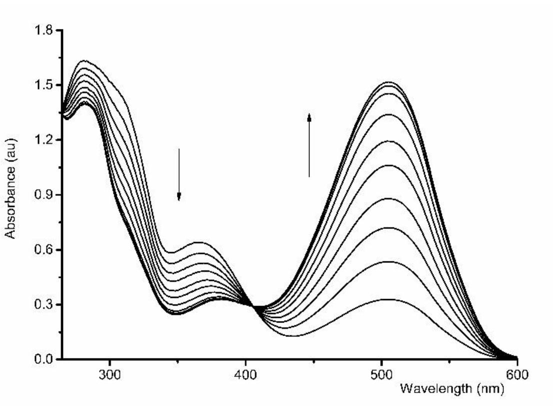

(b)

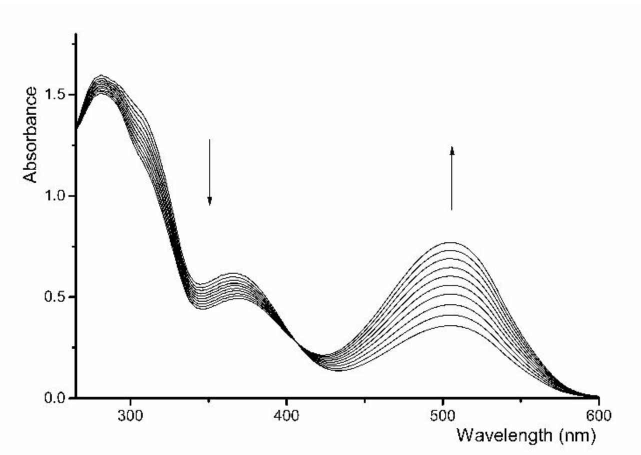

(c)

Figure S7. UV-vis spectral changes of compound **4** (4×10^{-5}mol·L^{-1}) upon the addition of various anions. a) F^- ($(0\text{-}8.0)\times10^{-5}$ mol·L^{-1}, b) AcO^- ($(0\text{-}6.4)\times10^{-5}$ mol·L^{-1}, c) HS^-($(0\text{-}6.4)\times10^{-5}$ mol·L^{-1}. Arrows indicated the direction of increasing anion concentration.

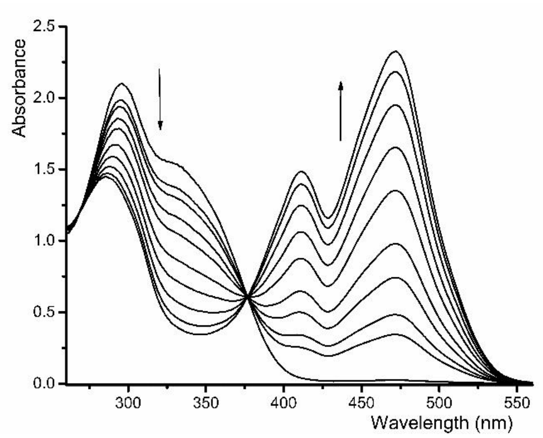

(a)

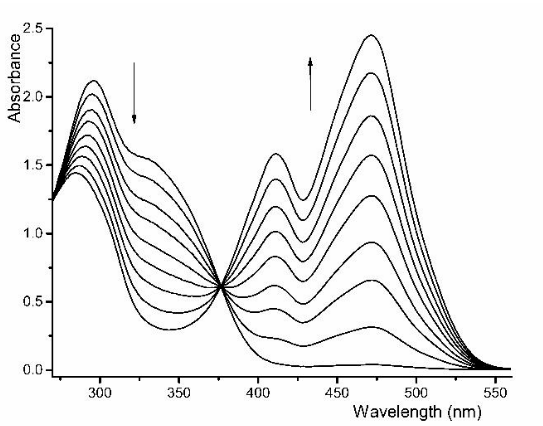

(b)

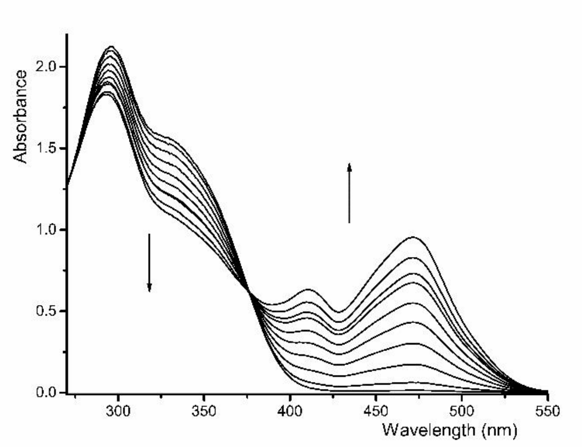

(c)

Figure S8. UV-vis spectral changes of compound **5** (4×10^{-5}mol·L^{-1}) upon the addition of various anions. a) F^- ((0-12.0)$\times10^{-5}$ mol·L^{-1}, b) AcO^- ((0-6.4)$\times10^{-5}$ mol·L^{-1}, c) HS^- ((0-17.6)$\times10^{-5}$ mol·L^{-1}. Arrows indicated the direction of increasing anion concentration.

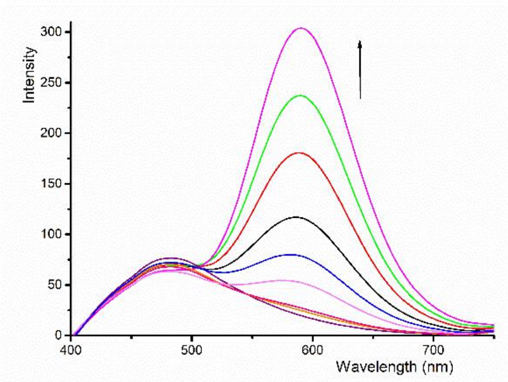

(a)

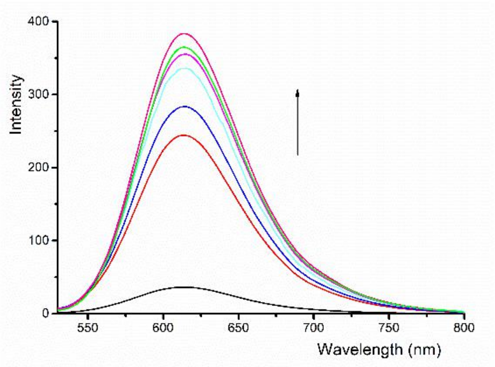

(b)

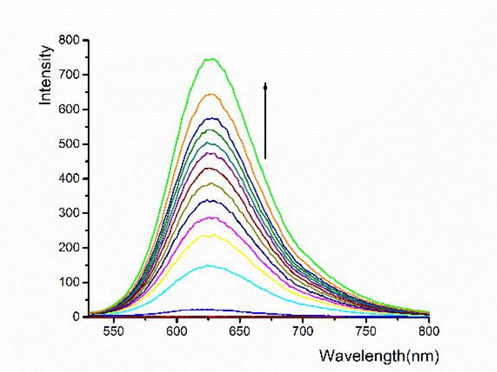

(c)

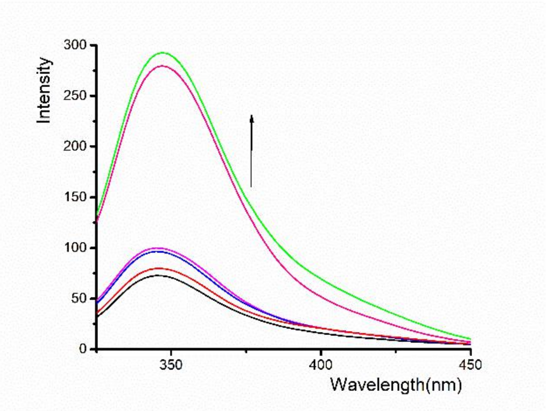

(d)

Figure S9. Fluorescence changes of compounds (4.0×10^{-5} mol·L^{-1}) with the addition of $H_2PO_4^-$. All spectra were recorded in DMSO solution, a) (λ_{ex} 382 nm, slit widths: 10 / 10 nm) of compound **1** up on the addition of $H_2PO_4^-$ ((0-208)$\times10^{-5}$ mol·L^{-1}), b) (λ_{ex} 510 nm, slit widths: 10 / 10nm) of compound **2** up on the addition of $H_2PO_4^-$ ((0-17.6)$\times10^{-5}$ mol·L^{-1})), c) (λ_{ex} 508 nm, slit widths: 5 / 5 nm) of compound **3** up on the addition of $H_2PO_4^-$ ((0-27.2)$\times10^{-5}$ mol·L^{-1})), d) (λ_{ex} 297 nm, slit widths: 10 / 10 nm) of compound **4** up on the addition of $H_2PO_4^-$ ((0-320)$\times10^{-5}$ mol·L^{-1})),

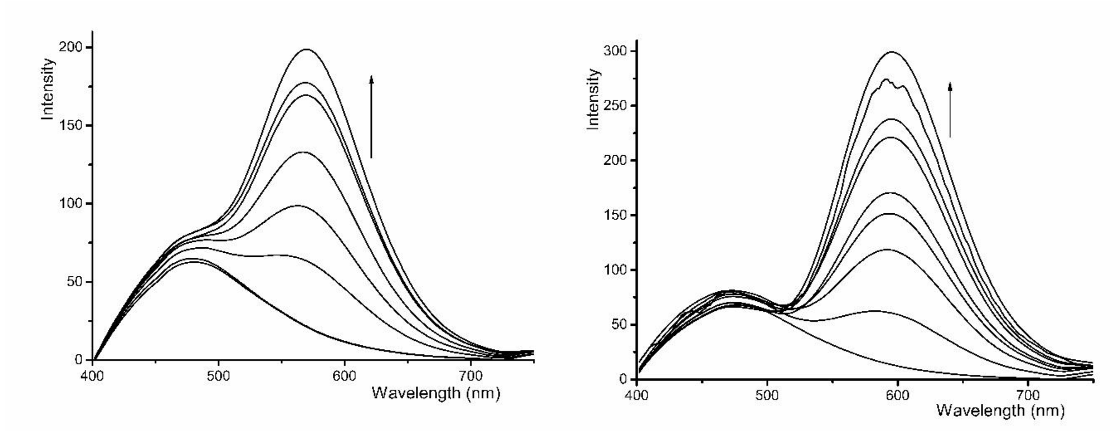

(a) (b)

Figure S10. Fluorescence titration (λ_{ex} 382 nm, slit widths:10nm/10nm) of compound **1** (4×10^{-5}mol·L^{-1}) with a) F^{-} ((0-160)$\times10^{-5}$ mol·L^{-1})), b) AcO^{-} ((0-64)$\times10^{-5}$ mol·L^{-1})) in DMSO solution.

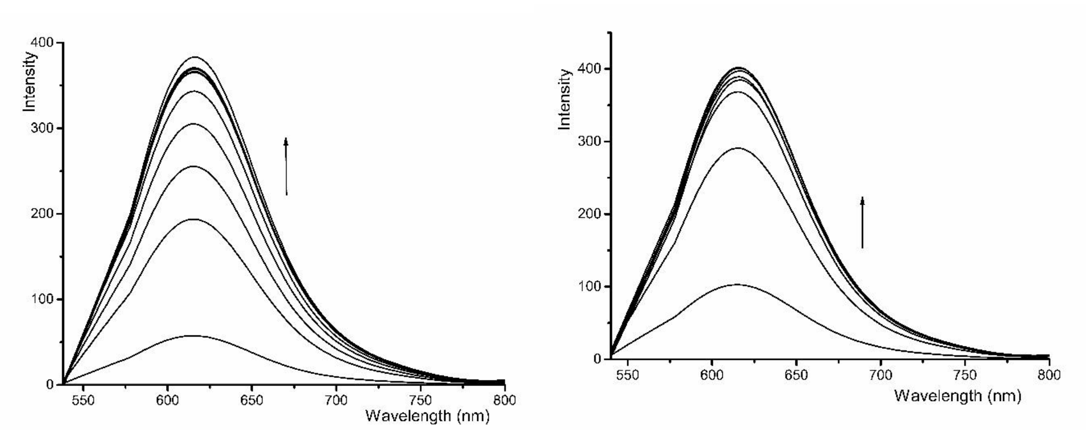

(a) (b)

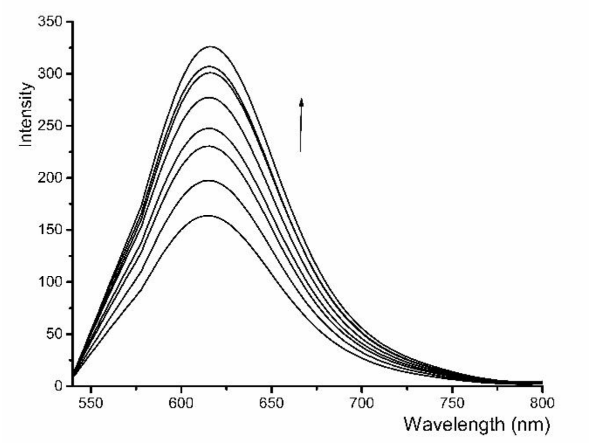

(c)

Figure S11. Fluorescence titration (λ_{ex} 510 nm, slit widths: 5nm/5nm) of compound **2** (4×10^{-5}mol·L^{-1}) with a) F^{-}

(($0-22.4)\times10^{-5}$ mol·L^{-1})), b) AcO$^-$ (($0-20.8)\times10^{-5}$ mol·L^{-1})), c) HS$^-$ (($0-57.6)\times10^{-5}$ mol·L^{-1})) in DMSO solution.

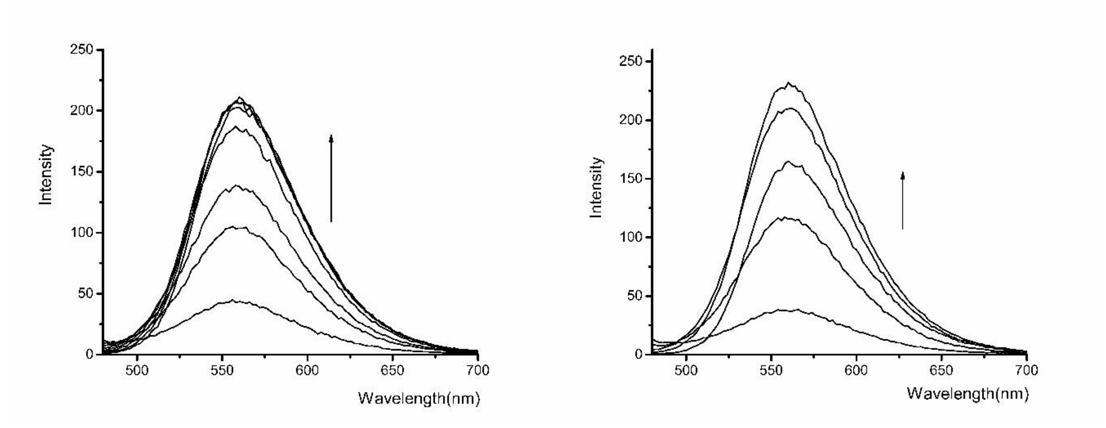

(a) (b)

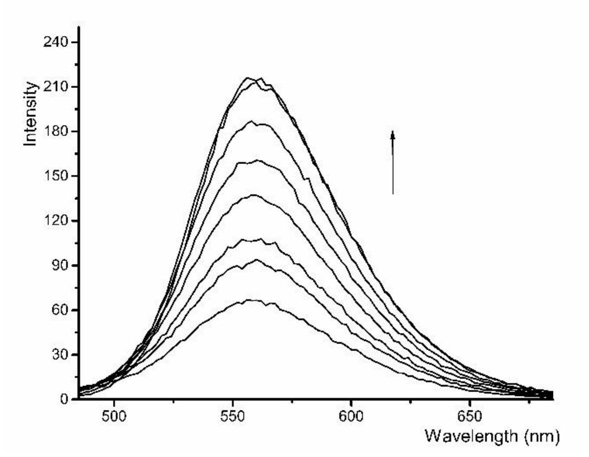

(c)

Figure S12. Fluorescence titration (λ_{ex} 412 nm, slit widths: 10nm/10nm) of compound **5** (4×10^{-5}mol·L^{-1}) with a) F$^-$ (($0-12)\times10^{-5}$ mol·L^{-1})), b) AcO$^-$ (($0-5.6)\times10^{-5}$ mol·L^{-1})), c) HS$^-$ (($0-17.6)\times10^{-5}$ mol·L^{-1})) in DMSO solution.

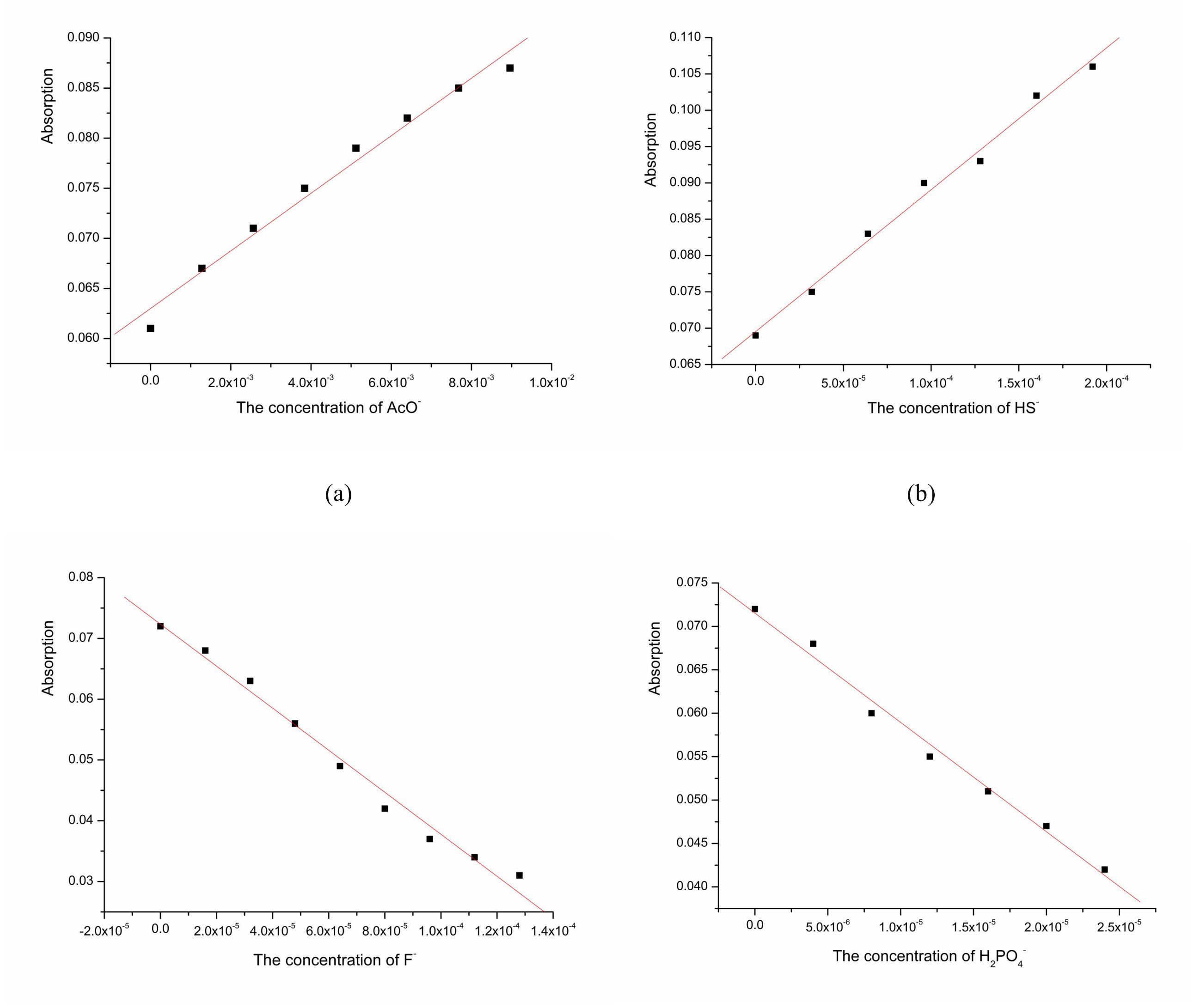

(a) (b)

(c) (d)

Figure S13. The linear relationship of UV-vis absorption at 470 nm of compound **5** in DMSO solution upon the addition of AcO^-(a), HS^-(b), F^-(c) and $H_2PO_4^-$(d). [compound **5**] = 2.0×10^{-6} mol • L^{-1} .

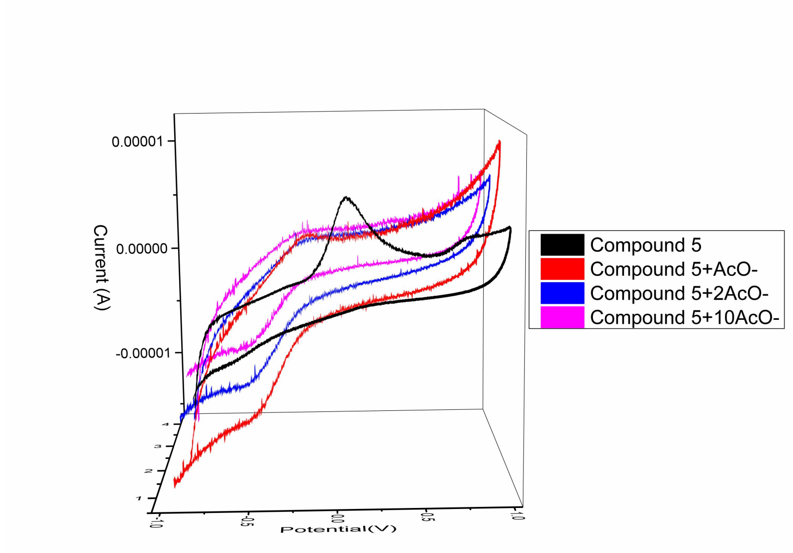

(a)

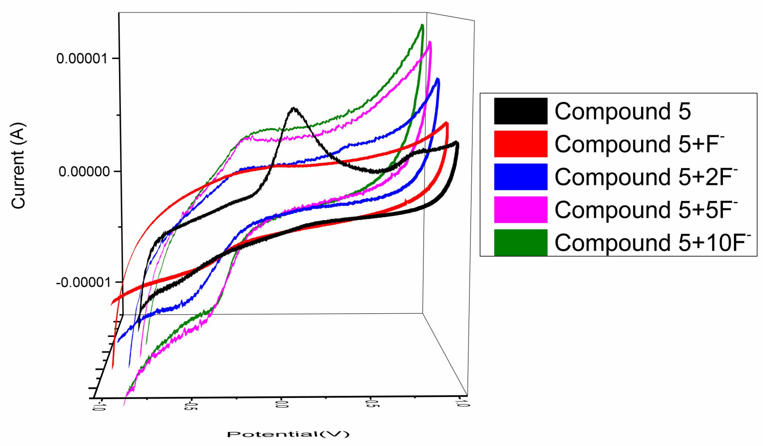

0.00001
0.00000
-0.00001
Current (A)
Potential(V)
Compound 5
Compound 5+F⁻
Compound 5+2F⁻
Compound 5+5F⁻
Compound 5+10F⁻

(b)

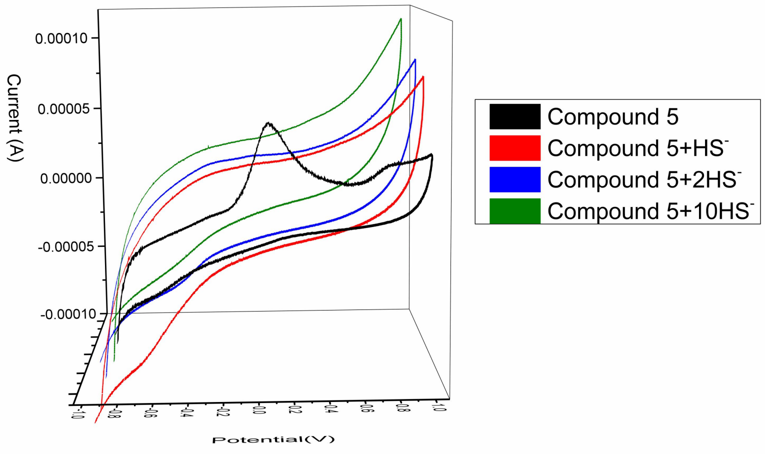

0.00010
0.00005
0.00000
-0.00005
-0.00010
Current (A)
Potential(V)
Compound 5
Compound 5+HS⁻
Compound 5+2HS⁻
Compound 5+10HS⁻

(c)

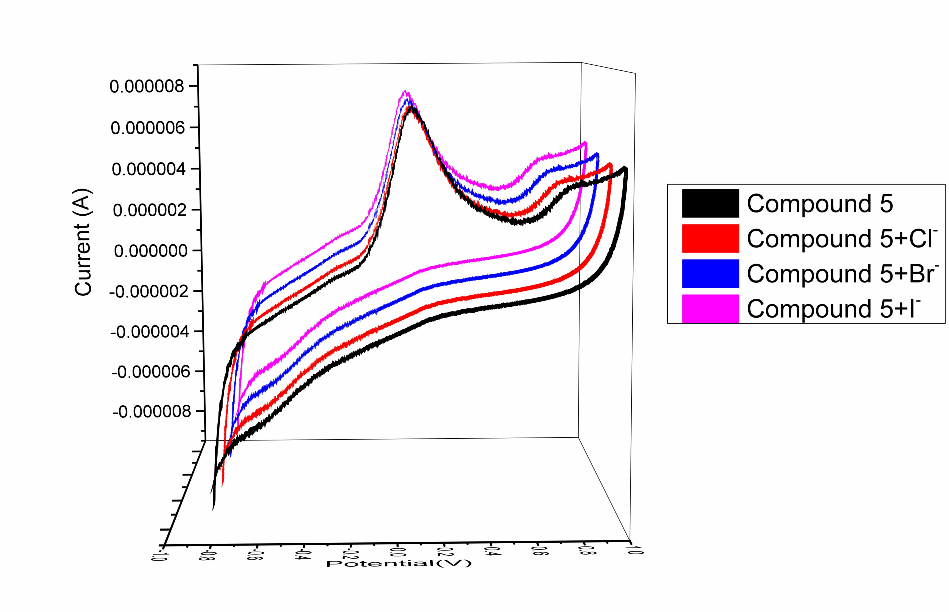

(d)

Figure S14. Cyclic voltammetry of compound **5** and Compound **5**-AcO$^-$(a), Compound **5**-F$^-$(b), Compound **5**-HS$^-$(c), Compound **5**-Cl$^-$/ Br$^-$, I$^-$ (d).

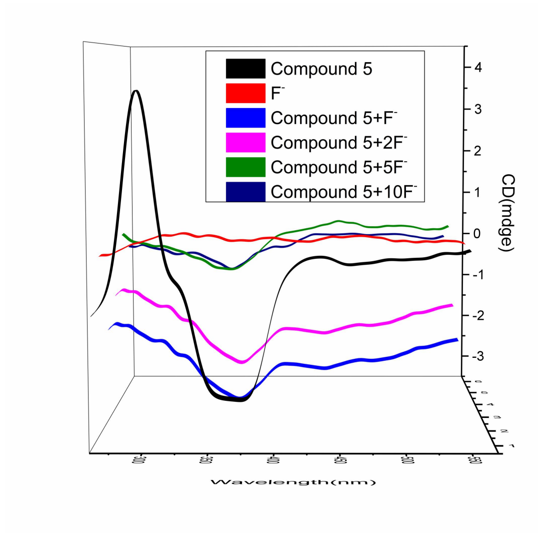

(a)

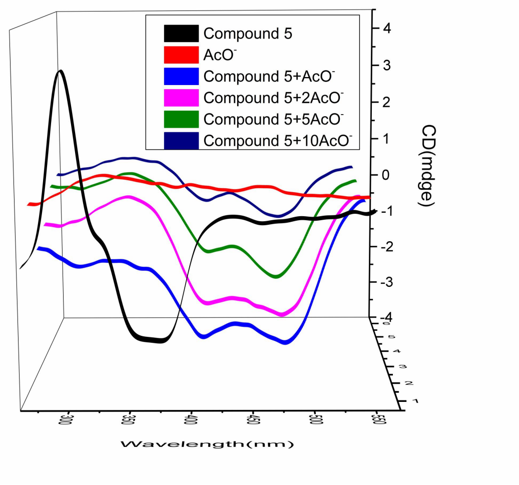

(b)

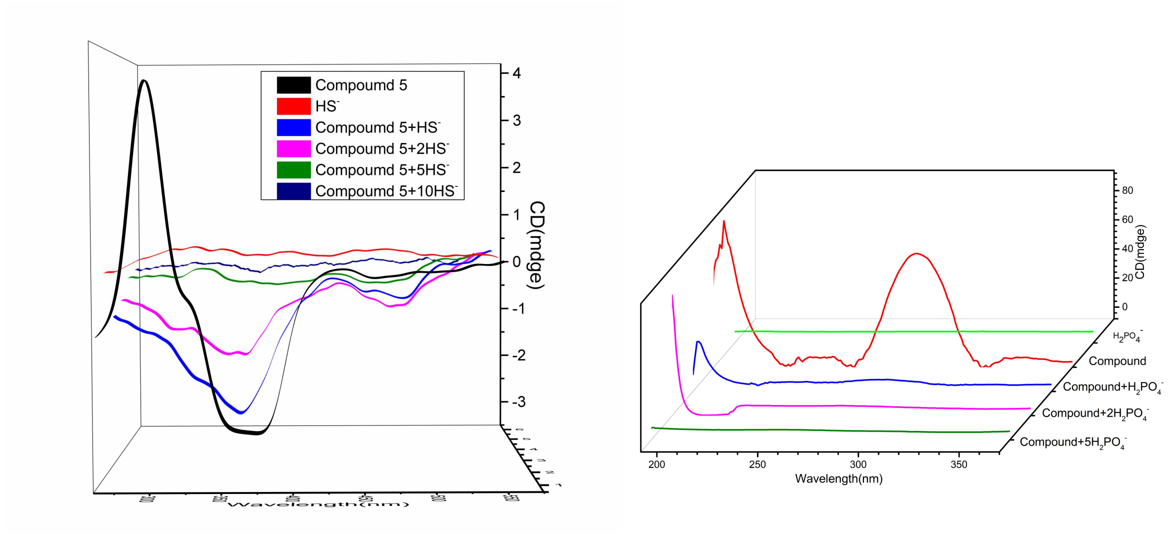

(c) (d)

Figure S15. The circular dichromatic spectra of compound **5** with F^-(a), AcO^-(a), HS^-(a), $H_2PO_4^-$ (D)in DMSO(a, b, c),and MeOH(d).

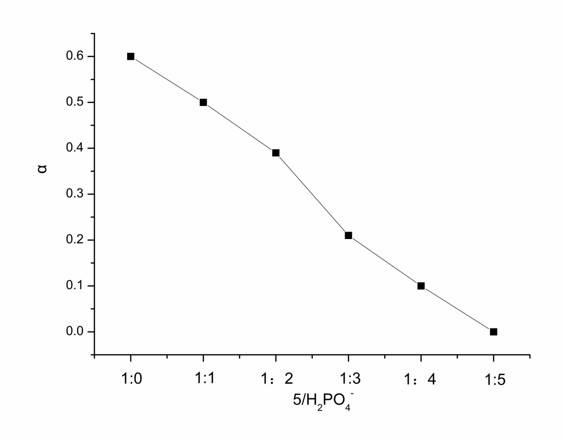

Figure S16. The change in optical rotation of compound **5** after binding with $H_2PO_4^-$ in MeOH.

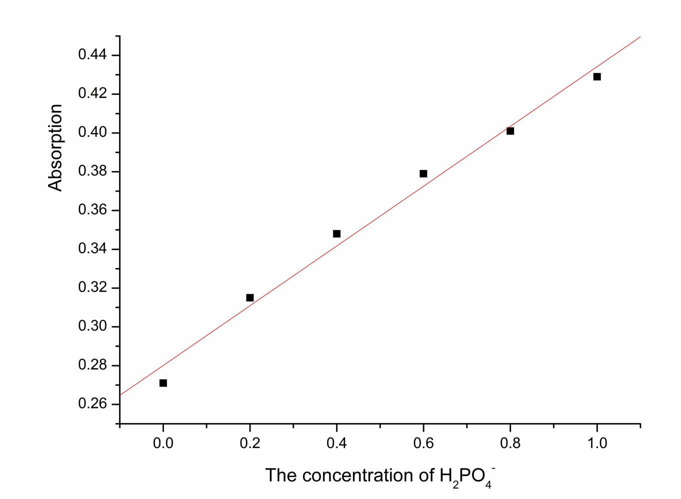

Figure S17. The linear relationship of UV-vis absorption at 470 nm of compound **5** upon the addition of $H_2PO_4^-$.

[compound **5**] = 4.0×10^{-6} mol • L^{-1} . [$H_2PO_4^-$] = 0-1.0 mg • mL^{-1} .

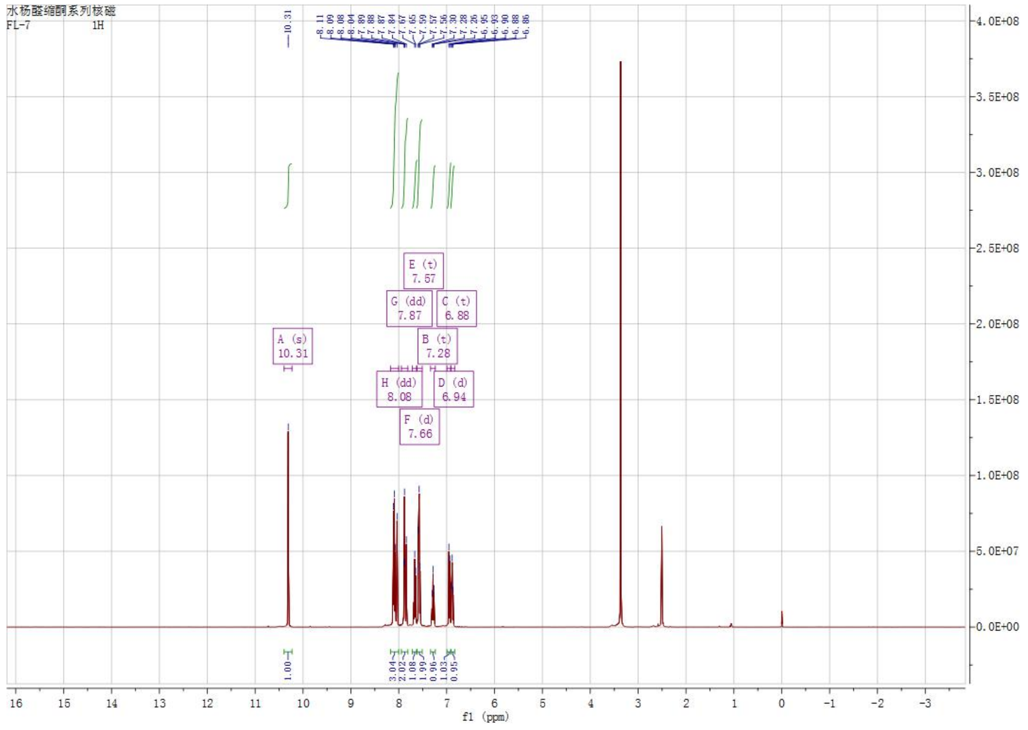

Fig. S18. ^{1}H NMR spectrum of compound **1**.

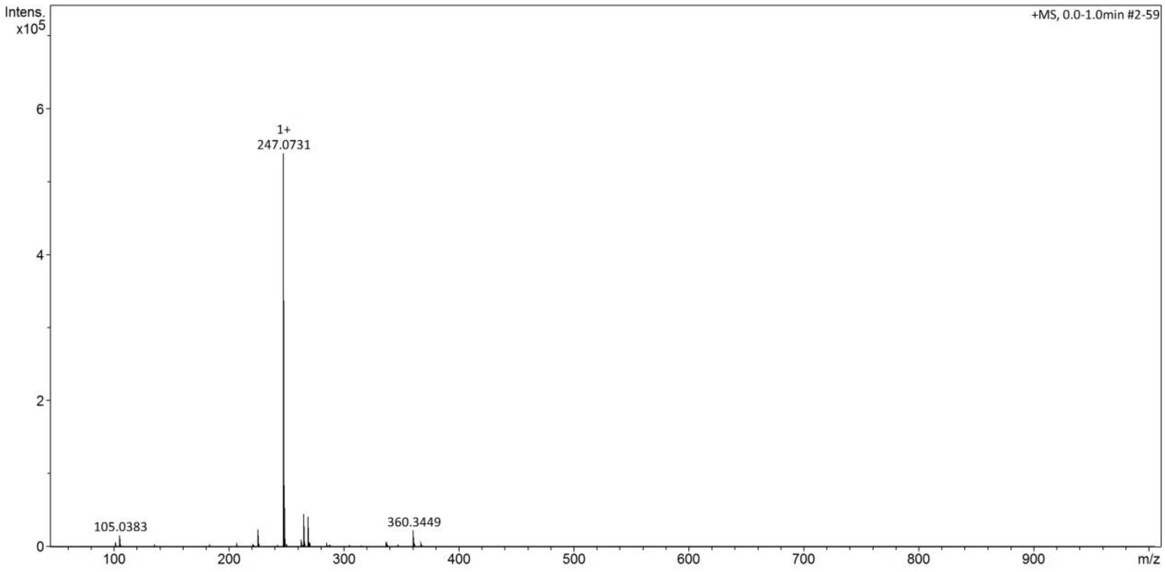

Fig. S19. ESI-HRMS spectrum of compound **1** ESI-HRMS (m/z): 247.0731 (M+Na)$^{+}$.

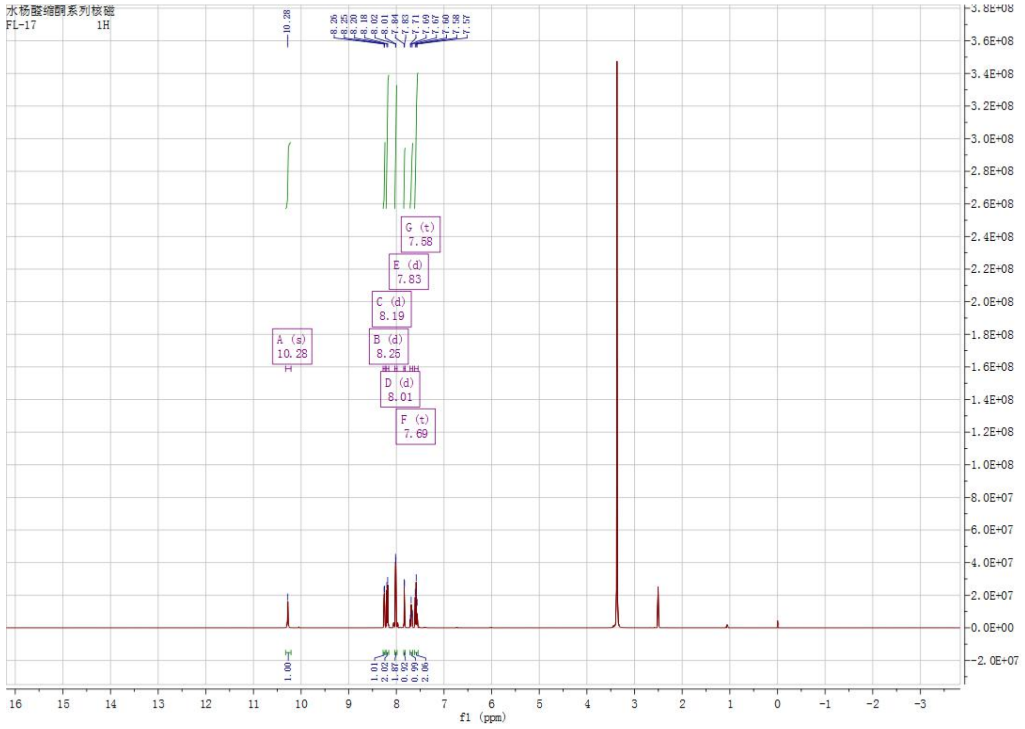

Figure S20. ^{1}H NMR spectrum of compound **2**

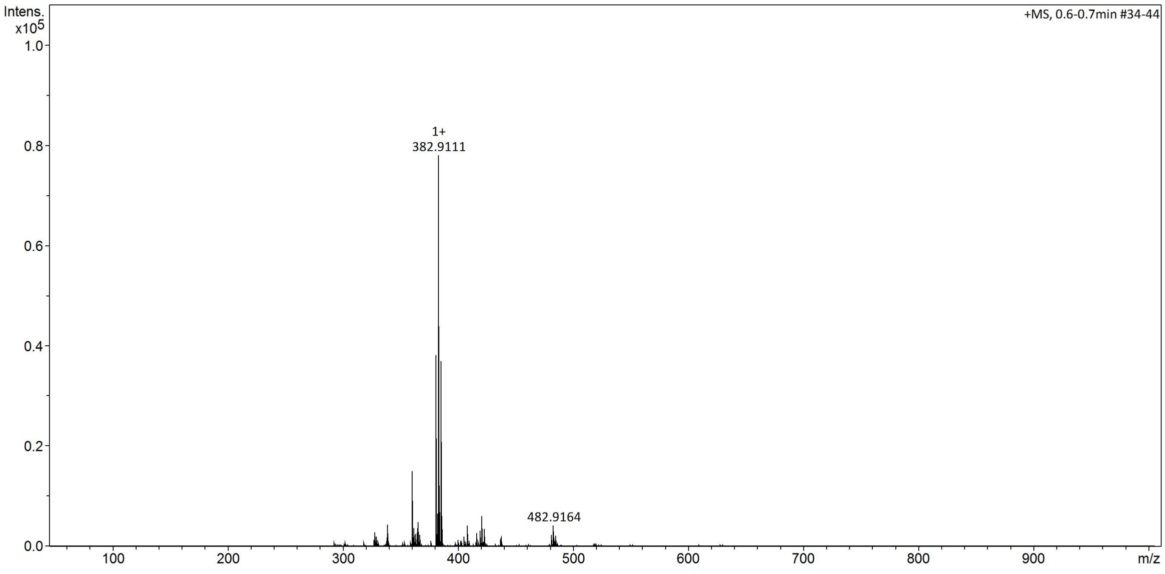

Figure S21. ESI-HRMS spectrum of compound **2** ESI-HRMS (m/z): 382.9111(M+H)$^{+}$.

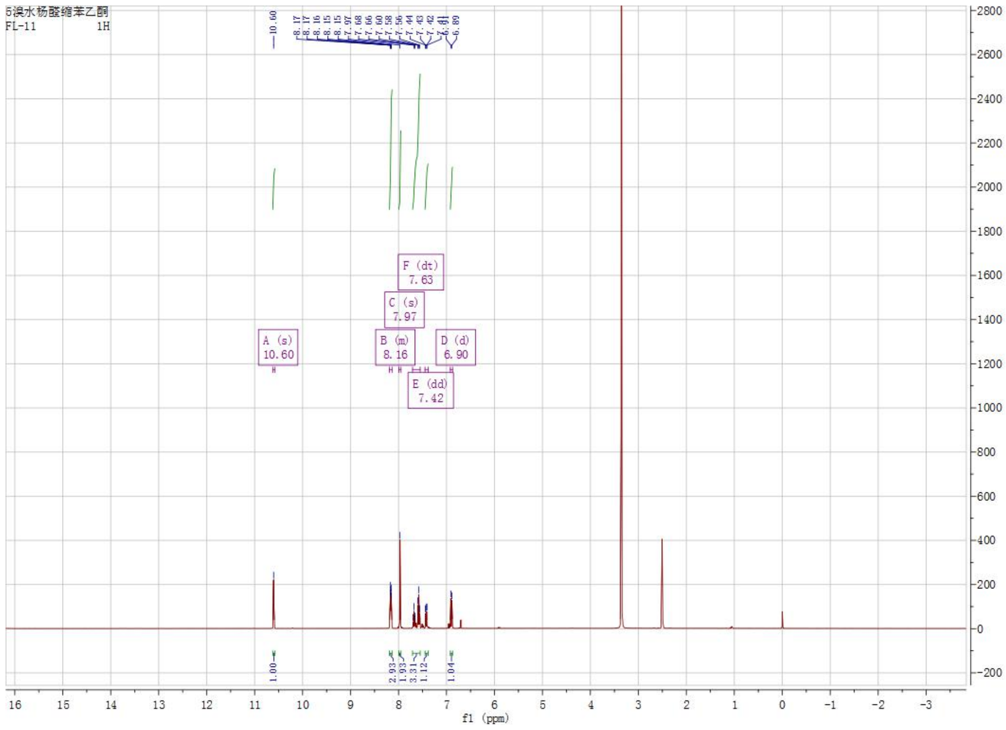

Figure S22. ^{1}H NMR spectrum of compound **3**.

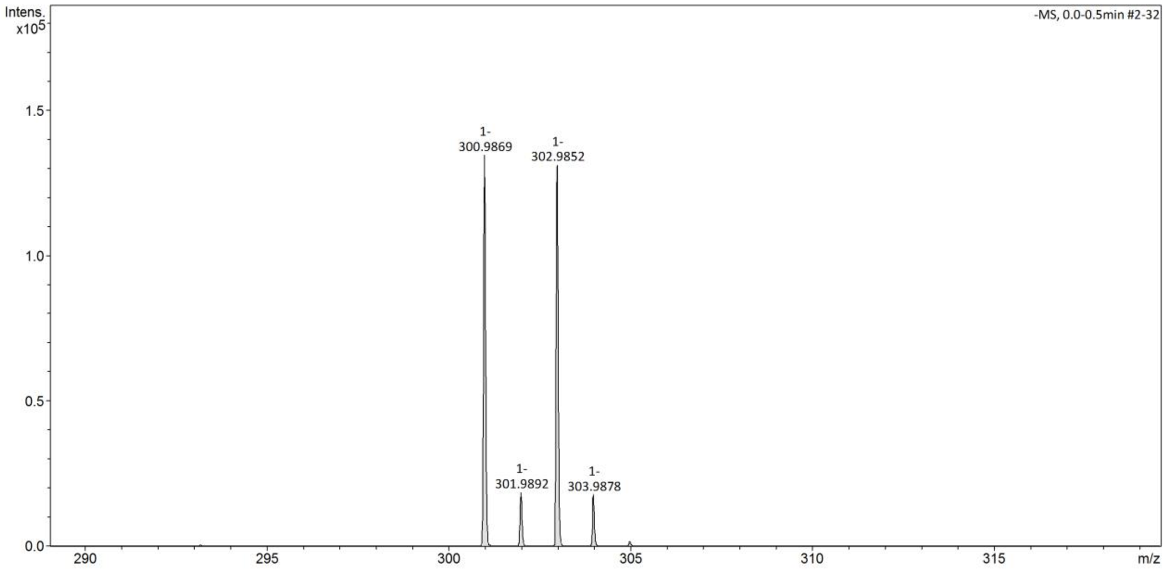

Figure S23. ESI-HRMS spectrum of compound **3** ESI-HRMS (m/z): 300.9869 (M-H)$^-$.

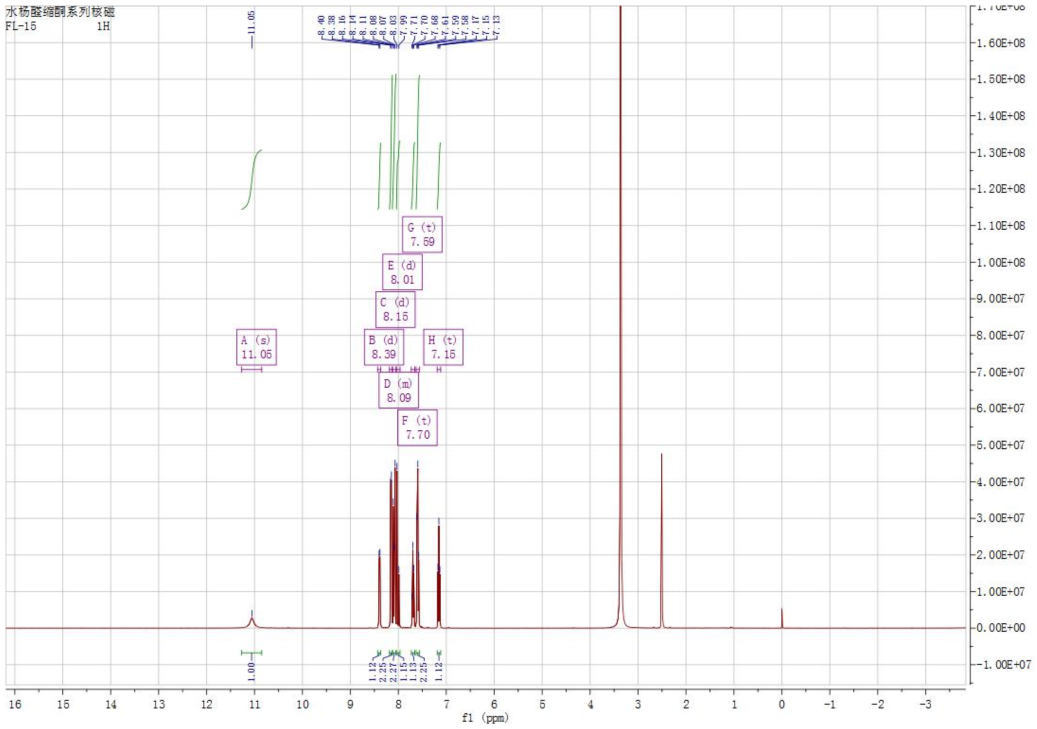

Figure S24. ^{1}H NMR spectrum of compound **4**.

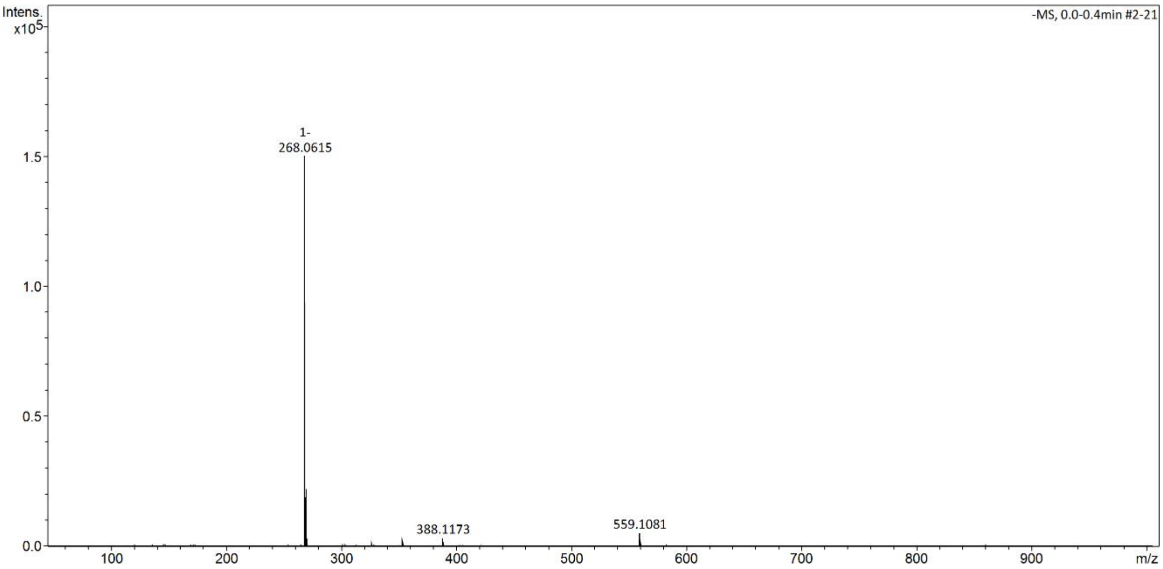

Figure S25. ESI-HRMS spectrum of compound **4** ESI-HRMS (m/z): 268.0615 (M-H)$^{-}$.

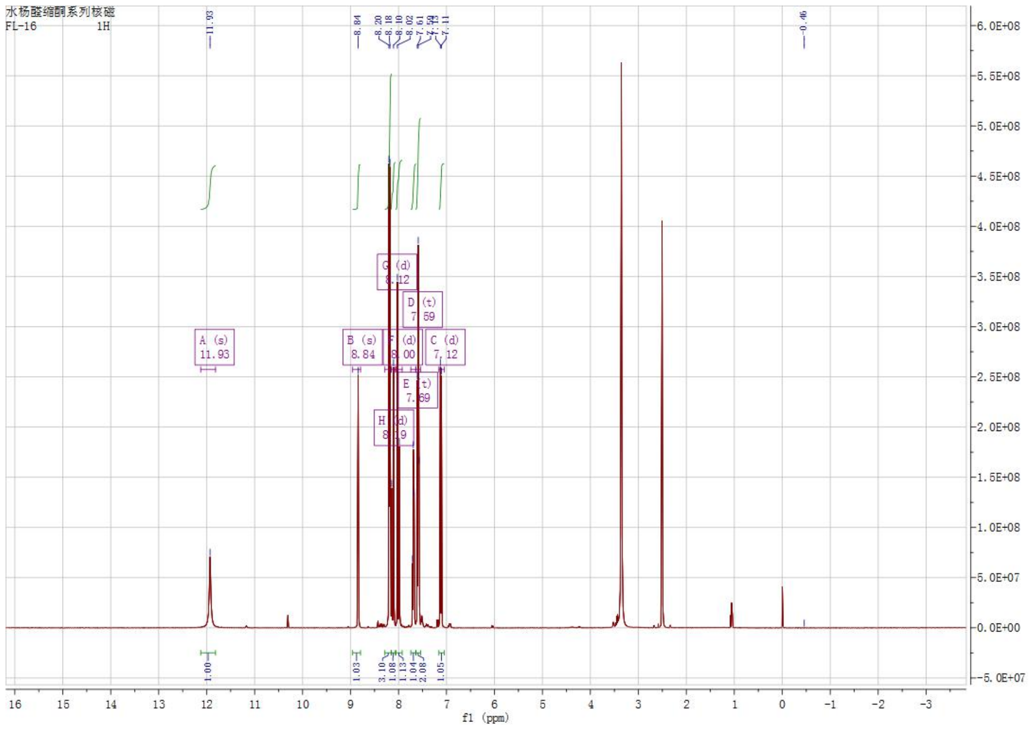

Figure S26. ^{1}H NMR spectrum of compound **5**.

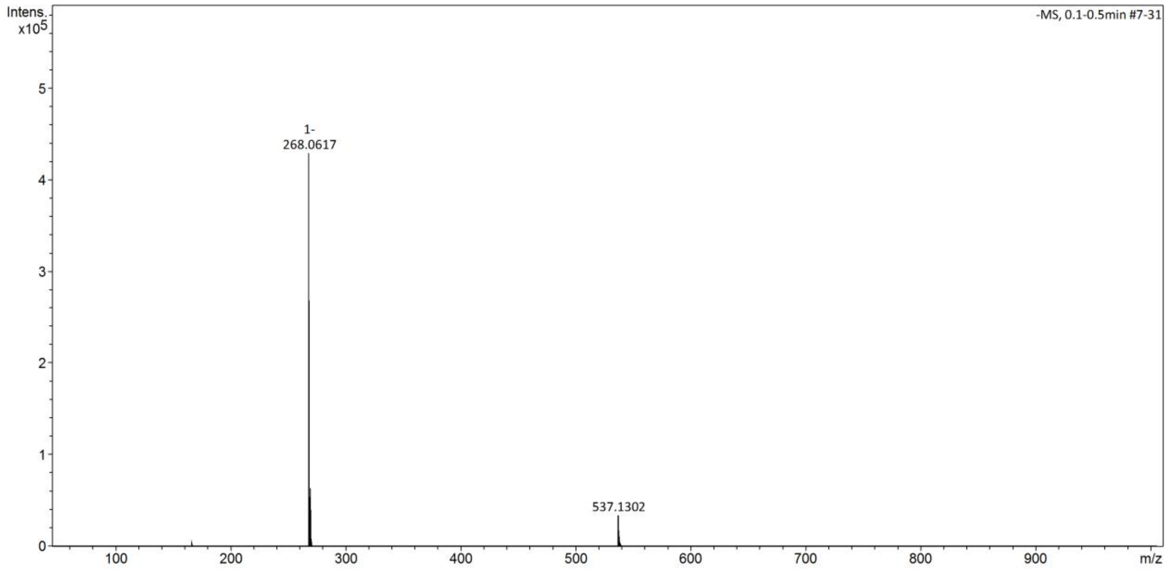

Figure S27. ESI-HRMS spectrum of compound **5** ESI-HRMS (m/z): 268.0617 (M-H)$^{-}$.

Table

Table S1. Crystal data and structure refinement for compound **1**

Compound	**1**	
Empirical formula	C15 H12 O2	
Formula weight	224.25	
Temperature	296(2) K	
Wavelength	0.71073 Å	
Crystal system	Monoclinic	
Space group	P 21/n	
Unit cell dimensions	a = 6.3587(3) Å	= 90°
	b = 12.8047(7) Å	= 91.1712(16)°
	c = 13.9659(7) Å	= 90°
Volume	1136.88(10) $Å^3$	
Z	4	
Density (calculated)	1.310 Mg/m^3	
Absorption coefficient	0.086 mm^{-1}	
F(000)	472	
Crystal size	0.506 x 0.257 x 0.187 mm^3	
Theta range for data collection	3.182 to 27.548°	
Index ranges	-8<=h<=8, -16<=k<=16, -17<=l<=18	
Reflections collected	17316	
Independent reflections	2620 [R(int) = 0.0984]	
Completeness to theta = 25.242°	99.9 %	
Absorption correction	Semi-empirical from equivalents	
Max. and min. transmission	0.976 and 0.961	
Refinement method	Full-matrix least-squares on F^2	
Data / restraints / parameters	2620 / 0 / 155	
Goodness-of-fit on F^2	1.018	
Final R indices [I>2 sigma(I)]	R1 = 0.0532, wR2 = 0.1378	
Final R indices [I>2 sigma(I)]	R1 = 0.0829, wR2 = 0.1586	
Largest diff. peak and hole	0.170 and -0.203 e.$Å^{-3}$	

Table S2. Hydrogen bonds for compound **1** [Å and °]

D-H...A	d(D-H)	d(H...A)	d(D...A)	<(DHA)
O(1)-H(1)...O(2)#1	0.82	1.93	2.7390(16)	171.0

Symmetry transformations used to generate equivalent atoms: #1 -x+5/2, y+1/2, -z+1/2

Table S3. Comparison of bond lengths between X-ray diffraction crystal structure and theoretical optimization model structure of compound **1** [Å]

	X-ray	Optimal		X-ray	Optimal
O(1)-C(1)	1.3493	1.355	C(3)-H(3)	0.9300	1.100
O(1)-H(1)	0.8200	0.972	C(12)-C(13)	1.368	1.420
O(2)-C(9)	1.2296	1.208	C(12)-H(12)	0.9300	1.100
C(10)-C(15)	1.385	1.420	C(13)-C(14)	1.372	1.420
C(10)-C(11)	1.393	1.420	C(13)-H(13)	0.9300	1.100
C(10)-C(9)	1.487	1.517	C(4)-H(4)	0.9300	1.100
C(6)-C(5)	1.393	1.420	C(14)-H(14)	0.9300	1.100
C(6)-C(1)	1.408	1.420	C(2)-H(2)	0.9300	1.100
C(6)-C(7)	1.455	1.503	C(15)-C(14)	1.383	1.420
C(8)-C(7)	1.325	1.337	C(15)-H(15)	0.9300	1.100
C(8)-C(9)	1.467	1.517	C(5)-C(4)	1.373	1.420
C(8)-H(8)	0.9300	1.100	C(5)-H(5)	0.9300	1.100
C(2)-C(3)	1.365	1.420	C(7)-H(7)	0.9300	1.100
C(2)-C(1)	1.394	1.420	C(11)-C(12)	1.381	1.420
C(3)-C(4)	1.384	1.420	C(11)-H(11)	0.9300	1.100

Table S4. Comparison of angles between X-ray diffraction crystal structure and theoretical optimization model structure of compound **1** [°]

	X-ray	Optimal		X-ray	Optimal
C(1)-O(1)-H(1)	109.5	108.000	O(2)-C(9)-C(10)	120.30	123
C(15)-C(10)-C(11)	118.32	120	C(8)-C(9)-C(10)	119.14	115
C(15)-C(10)-C(9)	122.79	117.6	C(8)-C(7)-C(6)	126.45	120
C(11)-C(10)-C(9)	118.89	117.6	C(6)-C(7)-H(7)	116.8	120
C(5)-C(6)-C(1)	117.56	120	C(12)-C(11)-C(10)	120.34	120
C(5)-C(6)-C(7)	122.58	120	C(12)-C(11)-H(11)	119.8	120
C(1)-C(6)-C(7)	119.83	120	C(10)-C(11)-H(11)	119.8	120
C(7)-C(8)-C(9)	122.79	117.6	C(2)-C(3)-C(4)	120.67	120
C(7)-C(8)-H(8)	118.6	120	C(2)-C(3)-H(3)	119.7	120
C(9)-C(8)-H(8)	118.6	120	C(4)-C(3)-H(3)	119.7	120
C(3)-C(2)-C(1)	120.21	120	C(13)-C(12)-C(11)	120.67	120
C(3)-C(2)-H(2)	119.9	120	C(13)-C(12)-H(12)	119.7	120
C(1)-C(2)-H(2)	119.9	120	C(11)-C(12)-H(12)	119.7	120
O(1)-C(1)-C(2)	122.25	124.3	C(12)-C(13)-C(14)	119.66	120
O(1)-C(1)-C(6)	117.54	124.3	C(12)-C(13)-H(13)	120.2	120
C(2)-C(1)-C(6)	120.20	120	C(14)-C(13)-H(13)	120.2	120
C(14)-C(15)-C(10)	120.64	120	C(5)-C(4)-C(3)	119.36	120
C(14)-C(15)-H(15)	119.7	120	C(5)-C(4)-H(4)	120.3	120
C(10)-C(15)-H(15)	119.7	120	C(3)-C(4)-H(4)	120.3	120
C(4)-C(5)-C(6)	121.97	120	C(13)-C(14)-C(15)	120.38	120
C(4)-C(5)-H(5)	119.0	120	C(13)-C(14)-H(14)	119.8	120
C(6)-C(5)-H(5)	119.0	120	C(15)-C(14)-H(14)	119.8	120
O(2)-C(9)-C(8)	120.56	123			

Table S5. Results of the determination of $H_2PO_4^-$ in fertilizer

Fertilizer	Label main content ($mg·mL^{-1}$)	This method ($mg·mL^{-1}$)	Measured/Label (%)	Add ($mg·mL^{-1}$)	Recovery (%)
1	0.392	0.388	99.0	0.5	99.1
2	0.392	0.386	98.5	0.5	98.8
3	0.392	0.382	97.5	0.5	98.5
